# Emerging two-dimensional nanomaterial and its modifications for enhanced antiviral applications: a review

**DOI:** 10.1098/rsos.242179

**Published:** 2025-09-03

**Authors:** Raktim Chowdhury, Sirazam Munira Aishee, Nafisa Islam, Nirupam Aich, Shoeb Ahmed

**Affiliations:** ^1^ Department of Chemical and Biomolecular Engineering, University of Tennessee Knoxville College of Engineering, Knoxville, TN, USA; ^2^ Department of Chemical Engineering, University of Michigan-Ann Arbor, Ann Arbor, MI, USA; ^3^ Department of Chemical Engineering, Bangladesh University of Engineering and Technology, Dhaka, Dhaka Division, Bangladesh; ^4^ Department of Civil and Environmental Engineering, University of Nebraska-Lincoln, Lincoln, NE, USA

**Keywords:** 2D nanomaterials, antiviral, MXenes, nanocomposite

## Abstract

Highly resilient pathogens, especially viruses and antibiotic-resistant bacteria, present formidable challenges to public health due to their ability to evade conventional treatments. Traditional microbial disinfection methods, such as chemical deactivation and physical filtration, often fail to effectively neutralize viruses, thus leading to harmful by-products. In light of these limitations, there is a growing need for innovative solutions to address viral disinfection. Photocatalytic microbial disinfection has emerged as a promising approach, primarily explored for bacterial pathogens. However, its antiviral potential remains underinvestigated. Two-dimensional (2D) nanomaterials, with their unique physico-chemical properties, represent a breakthrough in photocatalytic technology, offering advantages such as high surface area, tunable optical characteristics and enhanced generation of reactive oxygen species (ROS). This review assesses the photocatalytic properties of emerging 2D materials—such as graphene, transition metal dichalcogenides (TMDs), graphitic carbon nitride (g-C_3_N_4_), black phosphorus (BP) and MXenes—focusing on their potential for antiviral applications. While much of the current research emphasizes antibacterial activity, this review explores how functionalization, doping and composite formation of these materials could enhance their antiviral capabilities, offering a novel avenue for combating viral pathogens and addressing global health challenges.

## 1. Introduction

Infectious microbes, particularly viruses and antibiotic-resistant bacteria, present a formidable challenge in today’s world. These pathogens have evolved mechanisms to resist conventional treatments, making infections harder to cure and control [1]. Viruses, with their rapid mutation rates, continually adapt, outpacing vaccine development and antiviral therapies [2]. Meanwhile, antibiotic-resistant bacteria emerge due to overuse and misuse of antibiotics in healthcare and agriculture, threatening to render many of the currently used antibiotics as ineffective [3]. This situation necessitates an urgent, multifaceted approach to combat these threats. It involves developing new, more effective treatments and vaccines, implementing better infection control measures and promoting responsible antibiotic use. Moreover, global collaboration in surveillance and research is vital to understanding and responding to these evolving threats, ensuring public health preparedness against potentially devastating outbreaks [4].

Traditional microbial treatment methods, including physical removal through adsorption or filtration and chemical deactivation using disinfectants like chlorine, chlorine dioxide, ozone and ultraviolet (UV) radiation, often fail when it comes to viruses due to the small size and unique surface characteristics of the infectious agents. Techniques like granular activated carbon adsorption, while efficient at removing protozoan cysts and bacteria, fall short against viruses [5]. On the other hand, although chlorine effectively inactivates viruses, it increases the likelihood of producing disinfection by-products that have the potential to be mutagenic and carcinogenic [6,7]. Similarly, ozone and chlorine dioxide are potent against viruses but require on-site production and can also lead to undesirable by-products such as chlorite, chlorate and bromate [8]. To reduce these by-products, there has been a shift towards employing UV light for disinfection. Nevertheless, this approach faces its limitations, as certain viral strains, such as adenoviruses and rotaviruses, show high resistance to UV radiation, resulting in increased energy consumption and operational costs. Although water disinfection through solar-powered devices can be environmentally friendly, the devices are difficult to sustain in less developed areas as they may not fully inactivate viruses, indicating the need for prolonged treatment [9]. These limitations highlight the urgency for innovative microbial treatment methods to effectively combat viral pathogens and ensure broader safety.

The field of photocatalytic microbial disinfection has witnessed a notable surge in research and application over the last 10 years, with an emphasis on combating bacterial pathogens [10–14]. Despite this progress, only a few studies published so far explicitly target viruses, and fewer than half of those test true human pathogens rather than bacteriophage surrogates [15]. This leaves a clear research gap at the intersection of two-dimensional (2D) nanomaterials and antiviral photocatalysis. Viruses differ from bacteria in three definitive ways: (i) they are an order of magnitude smaller (20–300 nm versus greater than or equal to 500 nm), (ii) they carry either RNA or DNA protected by a protein capsid, and (iii) many clinically relevant species possess a phospholipid envelope that is highly susceptible to oxidative burst [16]. These structural features are susceptible to light-driven disinfection kinetics involving photocatalysts. These photocatalyst-based reactions can generate reactive oxygen species (ROS) at the virus–catalyst interface faster than either viral self-repair or the host-cell entry of viruses. Consequently, there is a pressing need for rigorous antiviral studies within the photocatalytic framework to understand and optimize this method for virus eradication.

In this evolving landscape of antimicrobial materials, 2D nanomaterials stand out as a superior alternative to conventional nanoparticles and three-dimensional nanostructures. These materials offer a plethora of advantages such as a high surface-to-volume ratio, robust mechanical properties, and exceptional electronic and optical characteristics that enhance photocatalytic performance. The broad surface area of 2D nanomaterials provides increased active sites for photocatalytic reactions, which can lead to more efficient interaction with microbial targets, including viruses. Furthermore, the unique physico-chemical properties of 2D nanomaterials can be leveraged to fine-tune their interaction with light, allowing for the generation of ROS under visible light and even near-infrared (NIR) illumination. This adaptability not only amplifies their disinfection capabilities but also broadens the scope of their applicability to various environmental conditions, thereby offering a cutting-edge approach to antiviral disinfection [17–21].

Graphene, as the forerunner of 2D nanomaterials, has sparked considerable interest for its potential in antimicrobial activity and photocatalytic applications aimed at bacterial and viral deactivation [17,22–25]. Its unique structure confers certain advantages as a photocatalyst, such as high surface area and excellent conductivity, which facilitate the generation and transfer of electron–hole pairs essential for photocatalysis. However, the absence of a band gap in graphene facilitates rapid recombination of photogenerated electron–hole pairs, reducing its efficiency for a prolonged period. In addition, the toxicity of graphene limits its biomedical applications [26–28].

The growing family of 2D nanomaterials offers a diverse palette of properties that can potentially address the limitations of graphene [29,30]. For instance, transition metal dichalcogenides (TMDs) and graphitic carbon nitride (g-C_3_N_4_) exhibit band gaps that enable them to harness visible light more effectively than graphene [31,32]. Black phosphorus (BP) and boron nitride (BN) offer tunable electronic and thermal properties [33,34], while MXenes, with their metal-like conductivity and hydrophilic surfaces, expand the possibilities for photocatalytic design [35].

The antimicrobial potential of these emerging 2D materials has gained significant attention, as evidenced by recent research. In their review, Zhao *et al.* focused on the photo-induced antibacterial effects of graphene, TMD and BP-based 2D materials [36]. Similarly, a recent article by Bhattacharjee *et al.* briefly described the antibacterial activity of 2D MXenes and their advancements in this area [37]. O’Dowd *et al.*, on the other hand, summarized the photocatalytic degradation of bacteria using graphene-based composites [38]. Meanwhile, Liu *et al.* explored the detailed mechanisms by which these 2D materials generate ROS upon light irradiation, leading to bacterial disinfection [39].

This review highlights the emerging landscape of 2D materials, such as BN, BP, TMD, MXenes and graphitic carbon nitride, emphasizing their photocatalytic properties for microorganism disinfection. Furthermore, it explores how surface functionalization, doping and composite formation can enhance the antimicrobial potential of these materials. Although much attention has been given to their antibacterial capabilities, the primary intent of this review is to assess their effectiveness against viruses, offering insights into their broader applicability in antiviral treatments.

## 2. Mechanism of photocatalytic inactivation

In the process of photocatalysis, when light is applied, with an energy equal to or greater than the semiconductor’s band gap, it prompts an electron to move from its lower-energy valence band (VB) state to its higher-energy conduction band (CB) state [40]. Throughout this process, the generation of electron–hole pairs (e^−^ and h^+^) occurs, which further plays a crucial role in the formation of ROS [41]. Typically, electrons (e^−^) engage in the reduction process by producing superoxide species (O_2_^−^), while photogenerated holes (h^+^) split water molecules to create hydroxyl radicals (OH^•^) for the oxidation process. In some cases, singlet oxygen (O_2_^•^) formation has been detected [42].

**Figure 1.**
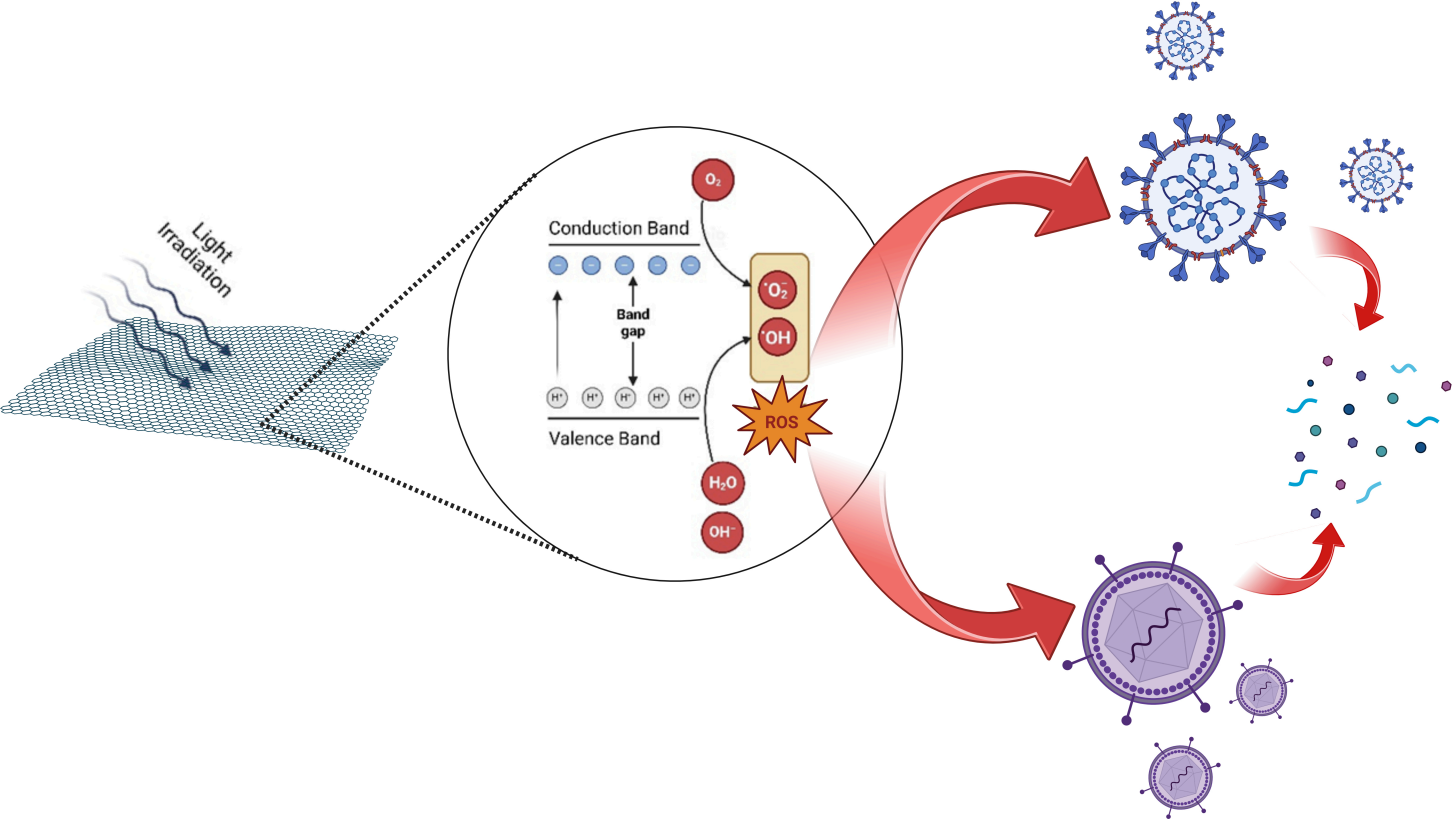
A schematic of the photocatalytic disinfection mechanism of virus with 2D materials.

This mechanism relies on three essential components: a photosensitizer, light and oxygen, all of which must be present at the same location [31,43–45]. When ROS levels exceed the cell’s buffering capacity, oxidative stress can occur, potentially leading to lipid peroxidation, oxidative protein carbonylation, enzyme deactivation and cell membrane permeability. The antiviral process is significantly influenced by oxidative stress mediated by ROS [46,47]. The overall process of this photocatalytic disinfection has been illustrated in figure 1.

### 2.1. Virus-specific pathways

The viral photocatalytic inactivation method targets three components: virus protein, nucleic acids (DNA/RNA) and viral lipids. The presence of viral lipids provides an extra site of attack for the enveloped viruses, rendering them more susceptible to viral inactivation. Certain photosensitizers (PS) have the ability to become incorporated within the viral DNA/RNA strands and induce oxidative reactions, resulting in the fragmentation of DNA, single-stranded breaks (SSB), or the formation of cross-links with proteins. Oxidative degradation of oxidation-sensitive amino acids in the protein backbone, such as methionine, tryptophan, histidine, etc., could result in structural alterations to the viral envelope proteins. Furthermore, the direct interactions between viral protein and PS might result in protein misfolding, which in turn can have an impact on viral function. It is important to mention that the presence of oxygen is not always obligatory for the production of O_2_^•^. Certain photosensitizers, such as psoralens, are capable of generating singlet oxygen (O_2_^•^) by a phototoxic mechanism that does not rely on oxygen [43,44].

### 2.2. Limitations of antibacterial data in predicting antiviral efficacy

While antibacterial data are often used as proxies for antiviral efficacy, this approach can be misleading due to fundamental differences in the inactivation mechanisms and kinetics of bacteria and viruses when exposed to ROS, particularly hydroxyl radicals (•OH). Bacteria, such as *Escherichia coli*, can be rapidly inactivated by •OH generated through advanced oxidation processes, achieving significant reductions in viability within minutes. By contrast, viruses often require longer exposure to achieve comparable levels of inactivation. For instance, during ozonation processes, the contribution of •OH to viral inactivation varies, with some viruses exhibiting resistance that necessitates prolonged treatment durations [48].

Additionally, bacteria possess enzymatic defence mechanisms against oxidative stress, notably catalases and peroxidases, which decompose hydrogen peroxide and mitigate ROS damage. These enzymes can complicate the quantification of ROS effects in bacterial systems, as they actively neutralize ROS, potentially leading to underestimations of oxidative damage. Viruses, lacking metabolic activity and such enzymatic defences, do not interfere with ROS quantification in the same manner, allowing for more straightforward assessments of oxidative inactivation [49].

Furthermore, the inactivation kinetics of bacteria and viruses differ notably. Bacterial inactivation often follows a log-linear pattern, indicating a consistent rate of kill over time. By contrast, viruses, particularly double-stranded DNA (dsDNA) viruses, frequently exhibit tailing in their inactivation curves, where the rate of inactivation decreases over time. This tailing effect suggests the presence of more resistant viral subpopulations displaying protective effects from the environment, which are not typically observed in bacterial kill curves [50,51]. The dynamics of virus inactivation are intricate and cannot be well represented by a single model, even when dealing with similar viruses [52,53].

Given these differences, it is imperative to conduct direct antiviral efficacy studies rather than extrapolating results from antibacterial data. The unique structural and functional characteristics of viruses necessitate specific testing to accurately assess the effectiveness of antiviral agents and treatments.

## 3. Antimicrobial effects of two-dimensional materials

### 3.1. Black phosphorus

BP is a metal-free semiconductor that exhibits a layer-dependent tunable band gap (0.3 eV, bulk; 2.0 eV, monolayer), strong absorption in UV and NIR regions, moderate carrier mobility, admirable biocompatibility and excellent biodegradability [33,54,55]. In BP, the phosphorus atoms have five valence shell electrons, forming a 3 s^2^ 3 p^3^ valence shell configuration. Each phosphorus atom bonds with three neighbouring phosphorus atoms via sp^3^ hybridized orbitals, and phosphorus atoms are positioned on a puckered honeycomb lattice structure. Each BP atom also has a lone pair, which makes phosphorus very sensitive to air [56,57].

#### 3.1.1. Black phosphorus as photocatalyst

Photocatalytic reactions with BP-based materials encompass four distinct types of connections: type I, type II, Z and S. Type I junctions have a significant rate of electron/hole (e^−^/h^+^) recombination, whereas type II junctions demonstrate effective separation of e^−^/h^+^. The junctions of the Z-scheme exhibit optimal electron–hole separation and possess a strong reduction capacity, with the OH^⦁^ radical playing a critical role. By contrast, the S-type junction preserves charge carriers with enhanced redox ability [58–60].

The water-exfoliated 2D BP nanosheets were shown to produce high singlet oxygen in the presence of O_2_ [61]. Interestingly, under UV irradiation, the BP nanosheets effectively generated OH^⦁^ radicals, while singlet oxygen radicals were the primary contributors under visible light [62–64].

#### 3.1.2. Antimicrobial application

**Figure 2.**
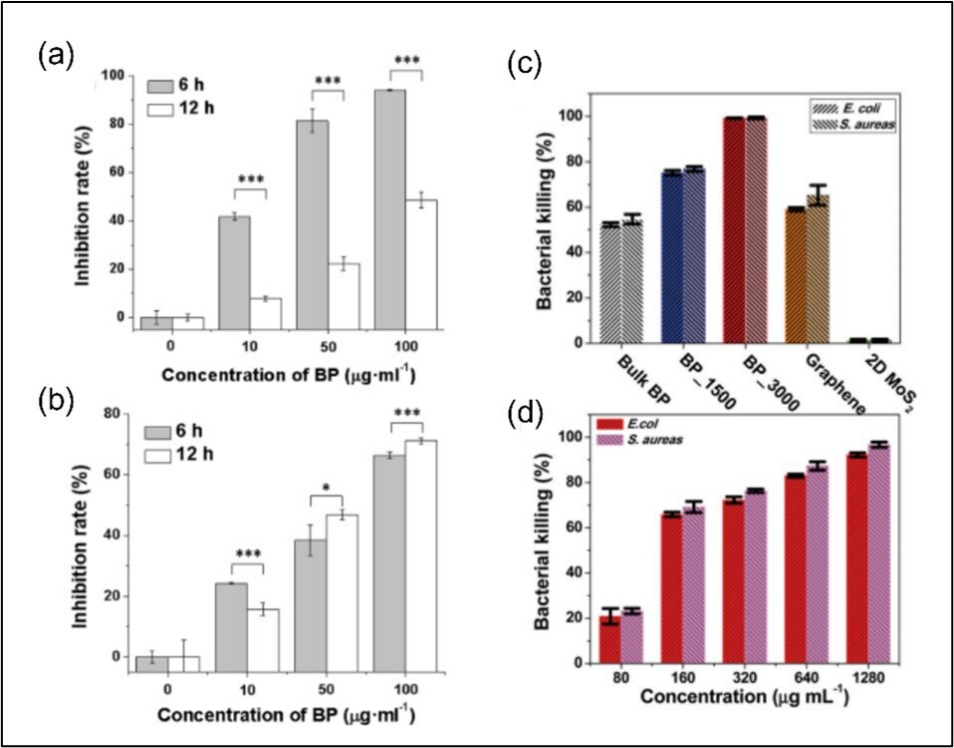
Antimicrobial activity of BP: the inhibition rate of BP nanosheets against (a) *E. coli* and (b) *B. subtilis* at 6 and 12 h. * and ***indicate *p* < 0.05 and *p* < 0.001, respectively [65]. (c) Bacterial killing ratios of bulk BP, BP_1500 and BP_3000 nanosheets, graphene and 2D MoS_2_ under the same experimental conditions [66]. (d) Bacterial killing ratio as a function of BP_3000 concentrations. The minimum inhibitory concentration (MIC) was found to be approximately 160 µg ml^−1^. The irradiation time in all cases was 3 min [66].

While evaluating the antibacterial activity of exfoliated BP nanosheets against gram-negative *E. coli* and gram-positive *Bacillus subtilis,* the researchers found that *E. coli* cells exhibited more susceptibility compared with *B. subtilis* cells after 6 h of UV exposure. However, after 12 h of exposure, the situation reversed (figure 2a,b) due to *E. coli*’s ability to self-heal its membrane. The ROS-dependent oxidative stress and membrane damage were reported to be a possible antibacterial mechanism of BP nanosheets [65]. Further research highlighted the effectiveness of BP nanosheets under visible light (660 nm), with a 10 min exposure resulting in the eradication of 99.3% of *E. coli* and 99.2% of *Staphylococcus aureus*. Without light irradiation, however, the disinfection rates dropped to 76.5% for *E. coli* and 69.7% for *S. aureus* [67]. On the other hand, in NIR region (808 nm), BP nanosheets demonstrated enhanced antibacterial properties (99.2% killing ratio) with minimal cytotoxicity compared with graphene (60%) and molybdenum disulfide (figure 2c) [66]. Notably, the antibacterial efficacy of the BP nanosheets was found to be layer-dependent, with thinner sheets exhibiting higher disinfection ratios [66].

#### 3.1.3. Composites and modifications

The photoactivity of BP can be significantly enhanced through structural modifications such as metal doping, surface functionalization and conjugation with other photosensitizers [68–71]. For instance, BP shows a layer-dependent band gap, and its moderate band gap can connect the energy gap between the zero band gap of graphene and the relatively large band gap of many transition metal dichalcogenides. By tuning the band gap and coupling with other metals, the BP adsorption spectrum can be broadened and its performance as a photosensitizer significantly improved [68–71].

Under UV irradiation, the BP/TiO_2_ hybrid material demonstrated photocatalytic activity comparable to that of TiO_2_, P25, and BP. However, when subjected to visible light, its disinfection efficiency against *E. coli* and *S. aureus* was remarkably superior, approaching complete bacterial elimination in 70 min, whereas the reduction achieved by TiO_2_, P25 and BP was limited to 20%. Additionally, the hybrid material maintained over 92% of its disinfection capability even after 15 cycles of use, in contrast to BP, which showed signs of degradation with repeated usage. This stability is attributed to the integration of Ti atoms into BP’s structure, enhancing its resistance to environmental factors [72].

**Figure 3.**
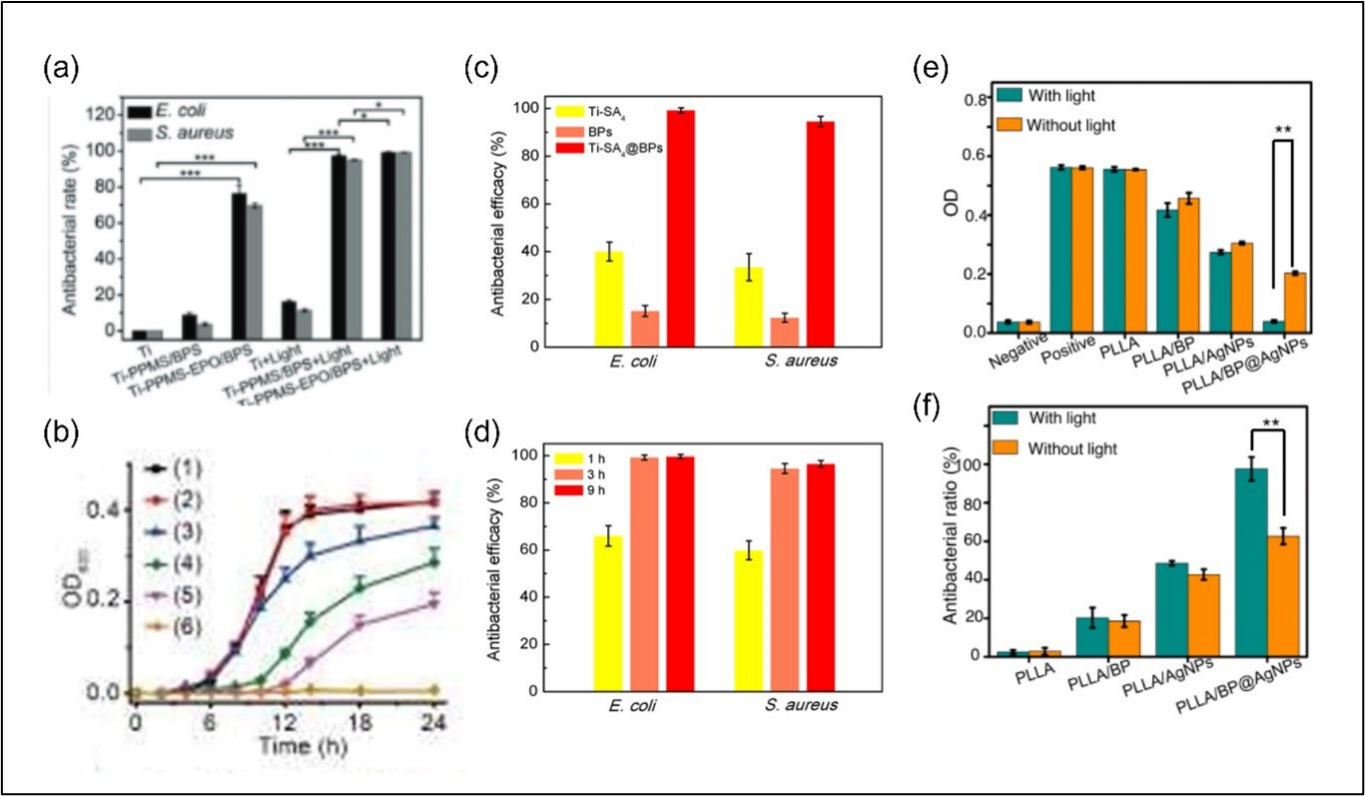
Antimicrobial activity of BP-based composites. (a) Antibacterial performance of Ti, Ti-PPMS/BPS, Ti-PPMS-EPO/BPS, Ti+light, Ti-PPMS/BPS+light, Ti-PPMS-EPO/BPS+light for *E. coli* and *S. aureus*, respectively. *n* = 3, *p < 0.05, ***p < 0.001 [67]. (b) The growth curves of MRSA bacteria after different treatments: (1) blank control, (2) NIR, (3) BP, (4) Ag@BP, (5) BP+NIR, (6) Ag@BP+NIR [74]. (c) Antibacterial activity of Ti-SA, BP and Ti-SA/BP against *E. coli* and *S. aureus* [75]. (d) Time-dependent antibacterial efficacy of Ti-SA 4@BPs [75]. (e) optical density and (f) bacterial inhibition rate of *S. aureus* on PLLA, PLLA/BP, PLLA/AgNPs and PLLA/BP@AgNPs after 24 h of culture [76].

BP/Ag nanocomposite shows photoactivity under irradiation with light of wavelength 300−2500 nm [73]. Combining the tunable band gap of BP and the localized surface plasmon resonance (LSPR) effect of AgNPs, the composite exhibits enhanced visible light absorption and photocatalytic antibacterial activity. The AgNPs act as electron acceptors, facilitating charge separation and minimizing electron–hole recombination. As a result, the hybrid material promotes ROS generation, effectively disrupting bacterial membranes, as well as damaging DNA and oxide proteins, with a 97% bactericidal rate under light exposure while also being cytocompatible (figure 3e,f) [76]. This biocompatibility makes the BP/Ag composite suitable for biomedical applications. In an *in vivo* study, it reduced the methicillin-resistant *S. aureus* (MRSA) count nearly to zero when applied to infection-associated tissue lesions in mice, underscoring its potential against antibiotic resistance. The underlying mechanism for this potent antibacterial action is concluded to be oxidative stress induced by NIR light [74].

A novel poly(4-pyridonemethylstyrene)-decorated BP photocatalyst with enhanced chemical stability showed outstanding photocatalytic performance with almost 100% disinfection efficiency for *E. coli* and *S. aureus* under only 10 min of illumination [67]. The incorporation of titanium aminobenzenesulfanato complexes (Ti-SA_4_) onto black phosphorus nanosheets (BPs) improved the antibacterial efficacy of BP from less than 20% to nearly 100% within 3 h with reduced protein synthesis and irreparable DNA damage (figure 3c). The strong P-Ti coordination between Ti-SA_4_ and BPs also enhanced the stability of BPs against oxidation [75].

Apart from these, several other BP-associated composites have highlighted their enhanced photocatalytic efficiency and superior ROS generation compared with base materials. The BP-Ag/TiO_2_ composite, for instance, outperformed Ag/TiO_2_ and GO-Ag/TiO_2_ under visible and NIR light [77]. Ternary composites combining AgBr, g-C_3_N_4_ and BP nanosheets showed significant improvement by promoting charge separation and reducing electron–hole recombination [59]. Other noteworthy developments include a metal-free gC_3_N_4_/BP composite modified with MoS_2_ [78], and a BP/porous g-C_3_N_4_-HKUST-1 photocatalytic membrane [69], both demonstrating high efficiency through effective interfacial charge separation. The ZIF-8-BP hybrid [79] and a double phosphorus-based BP/RP composite also exhibited remarkable photocatalytic degradation abilities [80]. Moreover, the CeO_2_/BP heterostructure’s type II heterostructure and high redox potential contributed to its exceptional performance under visible light [81]. Although these materials have not been tested for antiviral purposes yet, their photocatalytic capabilities suggest potential for such applications, underscoring the promising future of BP-based composites in photocatalysis.

### 3.2. MXenes

MXenes refer to a broad category of 2D transition metal carbides, nitrides or carbonitrides. The chemical formula employed is M_*n*+1_X_*n*_T_*x*_ (where *n* ranges from 1 to 4), with M representing an early transition metal such as Ti, V, Cr, Nb, Mo and others. X denotes the presence of carbon and/or nitrogen, while T*_x_* implies the surface termination groups, such as -F, -OH, -O and so forth [82,83]. Till now more than 30 MXenes have been synthesized and more than 100 MXene stoichiometries have been predicted by simulations, from which Ti_3_C_2_T_*x*_ is the most explored one [84,85].

Unlike mono transition metal MXenes, the double transition metal MXenes comprise two transition metal atoms, which can be classified into two categories [35]. The first category consists of solid solutions exhibiting a random distribution of transition metals in the M sites of the two-dimensional structure. The second category comprises ordered forms, which possess ordered structures either in-plane or out-of-plane [86]. They have hexagonal layered structures similar to their parent MAX phases [87,88]. The MAX phases are commonly represented by the M_*n*+1_AX_*n*_ formula, where *n* = 1, 2 or 3 and M, A and X represent an early transition metal, A-group (mostly groups 13 and 14) element and C and/or N, respectively [89]. The M-layers consist of interleaved A-group components, with X-atoms occupying octahedral positions. The bonding between the M and A elements is purely metallic, whereas the M−X bond encompasses the features of covalent, ionic and metallic bonds. However, it is worth noting that the M−A bonds are comparatively less stable when compared with the M−X bonds [90].

As most of the 2D MXene are metalloids and have a high free electron density, the ROS generation mechanism of metallic MXene is different from the other 2D materials. Liu *et al.* [91] describe the singlet oxygen ^•^O_2_ generation mechanism of MXenes to LSPR effect similar to that in Au or Ag nanoparticles. The energy transfer process that transfers photo-excited electrons from Ti_3_C_2_ to nearby oxygen molecules will cause the generation of ^•^O_2_. However, to date, only a few MXene species have been studied for photocatalytic ROS generation, and understanding the detailed mechanism requires further work [92].

#### 3.2.1. Two-dimensional MXenes as photocatalysts

MXenes exhibit remarkable optical characteristics, showcasing a broad absorption spectrum spanning from the UV to the IR range [93–95]. Based on the research findings, the Ti_3_C_2_T_*x*_ sheet not only has light absorption capabilities within the UV range of 300−500 nm but also demonstrates a broad absorption band in the range of 700−800 nm [96]. Zr_2_CO_2_ and Hf_2_CO_2_ also showed promising photocatalytic activity having a band gap ranging from 1.55 to 3.0 eV [97]. Additionally, these materials demonstrate remarkably high carrier mobility and substantial anisotropy, both of which contribute to the reduction of carrier recombination and the enhancement of photocatalytic activity. The findings from *ab initio* molecular dynamics (AIMD) simulations suggest that Zr_2_CO_2_ and Hf_2_CO_2_ possess stable, distinct two-dimensional structures when immersed in liquid water, hence establishing their stability as photocatalysts [98].

The characteristics and functionalities of MXene nanosheets are significantly influenced by surface chemistry due to their predominantly surface composition. Appropriate modification and adjustment of surface physico-chemical qualities will not compromise the initial 2D state and intrinsic attributes of MXenes. Instead, it can enhance the photocatalytic characteristics [99–102].

#### 3.2.2. Antimicrobial application

MXenes are emerging as potential candidates for inhibiting microbial growth due to their significant surface area, abundance of chemical functionalities that allow for chemical modifications and functionalization, and the presence of nucleophilic moieties like OH, F or O that facilitate the attachment of antimicrobial metallic adjuvants through coordination chemistry. Therefore, the use of these features of MXenes can lead to the development of more effective photo-sterilizers with improved performance [84]. One notable member of this family, Ti_3_C_2_T_*x*_, has demonstrated considerable potential in terms of its antibacterial properties, as evidenced by several studies [103–105].

**Figure 4.**
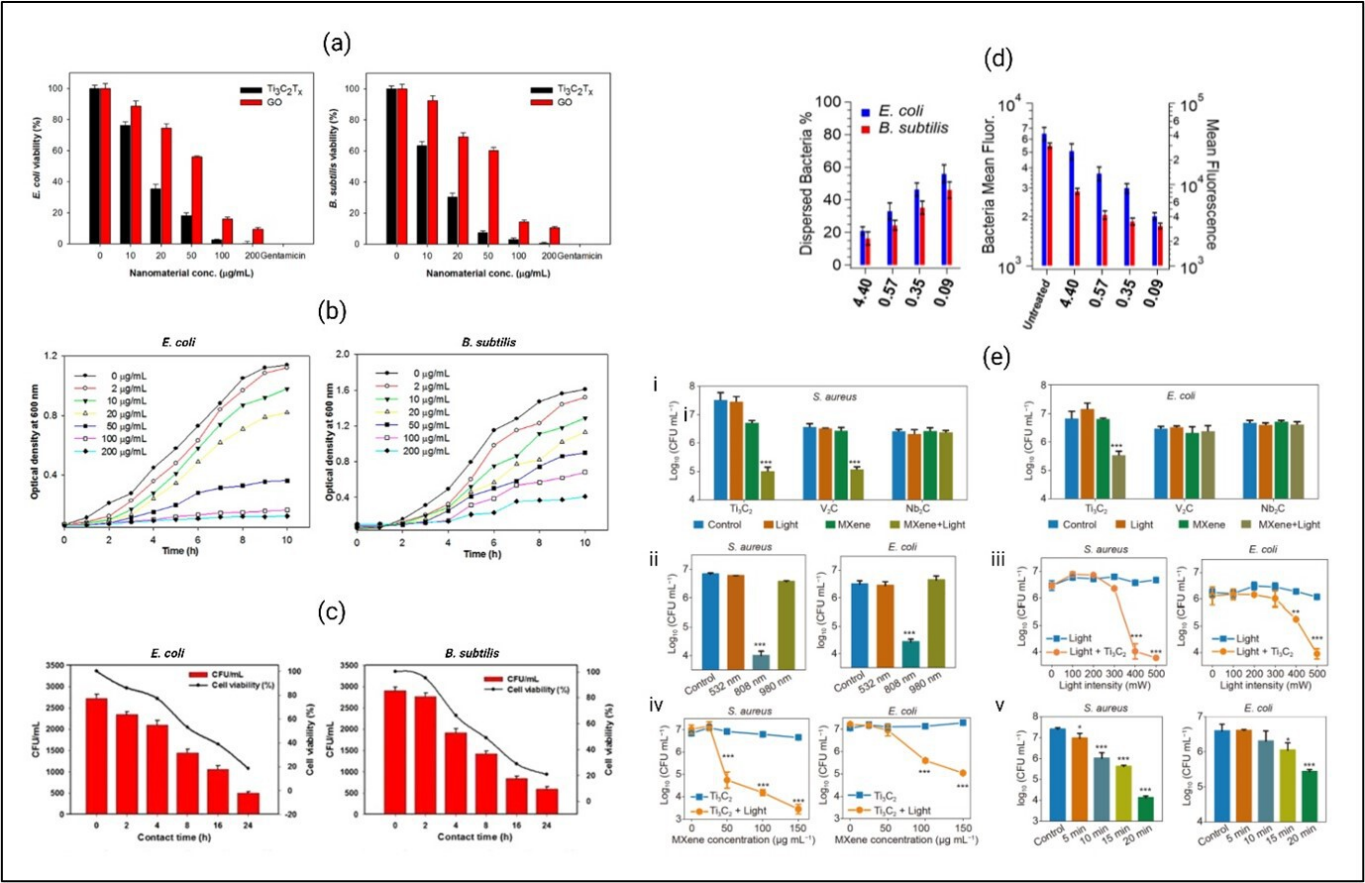
Antimicrobial activity of Mxenes. (a) Cell viability measurements of bacteria treated with Ti_3_C_2_T_*x*_ and graphene oxide (GO) of different concentrations (0−200 μg ml^−1^) in aqueous suspension. Survival rates were obtained by the colony forming count method. Gentamicin at concentration of 50 μg ml^−1^ was used as a positive control. Error bars represent the standard deviation [103]. (b) Bacterial optical density (OD) growth curve after being exposed to different Ti_3_C_2_T_*x*_ concentrations at 35°C for 4 h [103]. (c) Cell viability measurements of bacteria exposed to Ti_3_C_2_T_*x*_ membranes at different time intervals during 24 h of contact time. Survival rates were obtained by the colony forming count method as compared with that of the control polyvinylidene difluoride (PVDF) membrane [104]. (d) Antibacterial activity of various sizes of Ti_3_C_2_T_*x*_ MXene nanosheets against *B. subtilis* and *E. coli* investigated via flow cytometry (FC) analyses. Bacteria were treated with 100 μg ml^−1^ MXene nanosheets for 3 h in the dark. The percentage of MXene-induced dispersed bacteria and the bacteria mean fluorescence is quantified and depicted [106]. (e) Statistical analysis of bacterial concentration after treatment with different types of MXenes (i), different light wavelengths (ii), different light intensity (iii), different MXene concentration (iv), and different exposure times (v). Error bars represent standard deviation. *p < 0.05, **p < 0.01, ***p < 0.001 [107].

The single-layered exfoliated MXene (S-Ti_3_C_2_T_*x*_) performs significantly better than the MAX phase (Ti_3_AlC_2_) and few-layered unexfoliated (F-Ti_3_C_2_T_*x*_) MXene sheets against *B. subtilis* and *E. coli* (figure 4a,b) [103]. This highlights the importance of the thickness of the MXenes in their antimicrobial efficiency. The MXenes are also found to possess a greater antibacterial activity compared with graphene oxide (GO), and the inactivation mechanism consists of cell membrane rupture and ROS generation. Illustrated in figure 4c, a micrometre-thick Ti_3_C_2_T_*x*_ MXene-based antibacterial membrane can inhibit bacterial adhesion and biofilm formation on the surface of separation membranes [104]. However, the Ti_2_CT_*x*_ MXenes happen to lack antimicrobial properties against *Sarcina*, *S. aureus* and *Bacillus* sp*.* [108]. Similarly, the Ti_3_C_2_ MXene phase shows antimicrobial activity against *E. coli*, whereas Ti_2_C remains ineffective [109]. So, it is evident that the stoichiometry structure of the MXenes at the atomic scale has a significant impact on their bactericidal properties. The recent work of Arabi Shamsabadi *et al.* on Ti_3_C_2_T_*x*_ MXene nanosheets pointed out another interesting variable−lateral size. The colloidal solution of MXene sheets having smaller sizes exhibited a higher degree of destruction towards the bacterial cells [106].

The photothermal characteristics demonstrated by MXenes render them viable contenders for the development of photo sterilizers with exceptional performance capabilities [110]. Specifically, Ti_3_C_2_ has the potential to enable nearly complete conversion of photons to heat [111]. Furthermore, the hydrophilicity of the MXenes facilitates intimate contact with the sharp edges of the nanosheets, which ensures efficient heat transfer to the bacterial cells and thereby enhances antimicrobial activity [112]. As a result, the MXenes have exhibited effectivity against a total of 15 microbial strains, including those that are resistant to antibiotics, such as MRSA and vancomycin-resistant *Enterococci* (VRE) under the NIR irradiation at a wavelength of 808 nm [107]. Moreover, the photo-induced properties of Mo_2_C nanospheres in the NIR region show remarkable efficiency in both photothermal and photodynamic therapy, along with excellent biocompatibility [113].

Given the established antibacterial efficacy of 2D MXenes, researchers have extended their investigations to explore their antiviral properties. Both Ti_3_C_2_T_*x*_ and Mo_2_Ti_2_C_3_T_*x*_ have demonstrated significant antiviral activity against various strains of SARS-CoV-2, even at low concentrations. This antiviral effect is largely due to the strong interactions between viral proteins and the polar, negatively charged and redox-active surfaces of MXenes [85]. Apart from the physical properties of these materials, the genotypes and mutations have a significant role in determining the antiviral activity. According to the *in silico* and proteomic analyses using viral protein domains, Ti_3_C_2_T_*x*_ can interfere with the viral life cycle through multiple mechanisms including membrane trafficking, G protein-coupled receptor (GPCR) signalling and mitochondrial function, as well as disrupting cell surface [114]. Moreover, in the presence of oxygen and light irradiation, MXenes can disrupt the structure of the SARS-CoV-2 virus by generating ROS [115]. These exceptional photodynamic capabilities of MXenes hold the potential for treating virus-infected organs and developing highly effective non-touch sanitizers and sterilizers [116].

#### 3.2.3. Composites and modifications

**Figure 5.**
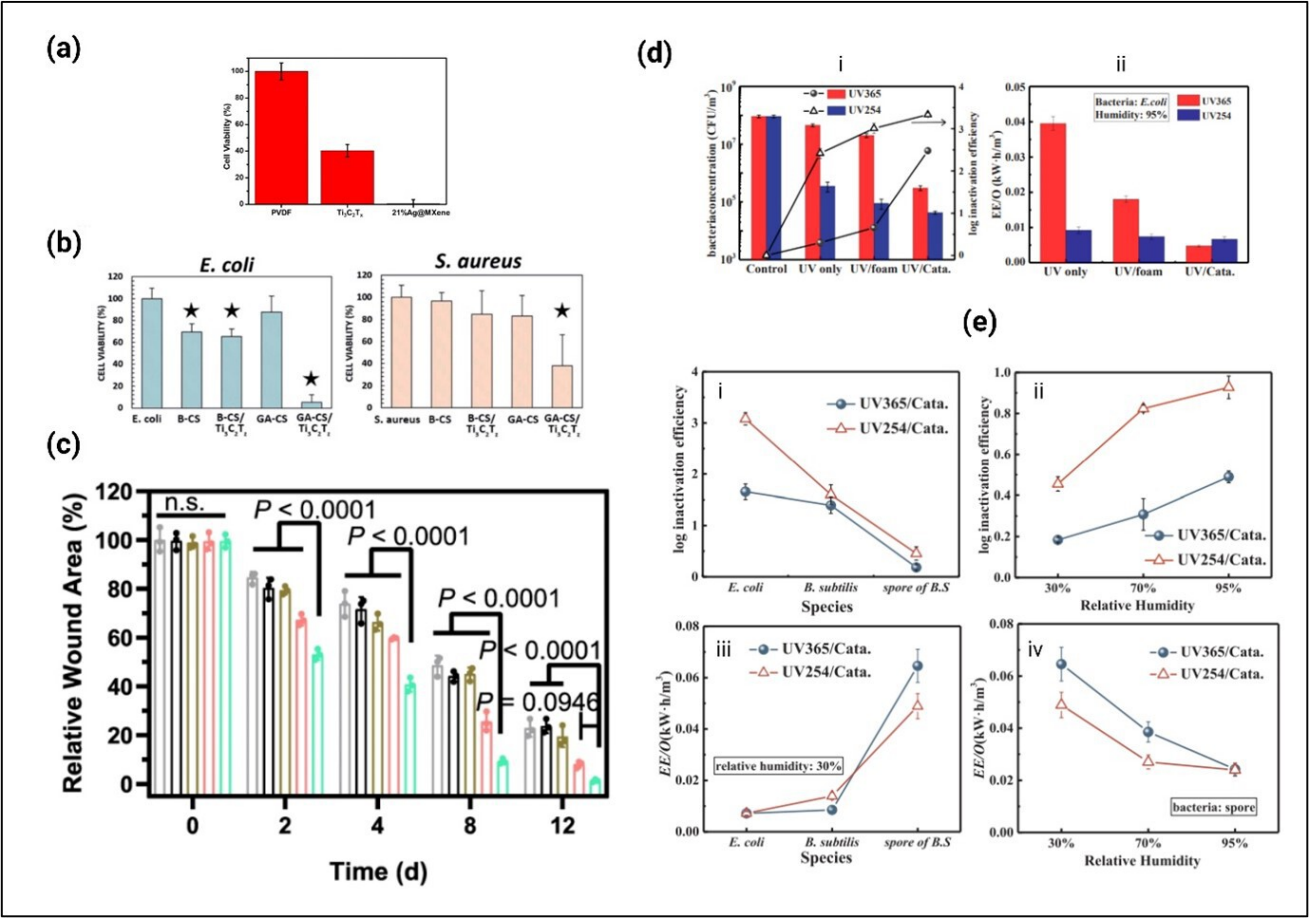
Antimicrobial activity of MXene-based composites. (a) Cell viability measurements of *E. coli* with PVDF (control), MXene (Ti_3_C_2_T_*x*_) and 21%Ag@MXene membranes [117]. (b) Antibacterial properties of electrospun Ti_3_C_2_T_*z*_ (MXene)/chitosan nanofibres against *E. coli* and *S. aureus*. B-X and GA-X indicate mats treated with NaOH and glutaraldehyde, respectively [118]. (c) The quantitative result of relative wound area on 2, 4, 8 and 12 d of control, 3 M wound dressing, Ti_3_C_2_T_*x*_, Bi_2_S_3_ and Bi_2_S_3_/Ti_3_C_2_T_*x*_−5 on *S. aureus* (10 μl, 1 × 10^8^ CFU ml^−1^) to infective wound healing *in vivo*. Grey circles indicate the group of Ctrl, black circles indicate the group of 3 M, brown circles indicate the group of Ti_3_C_2_T_*x*_, pink circles indicate the group of Bi_2_S_3_ and green circles indicate the group of Bi_2_S_3_/Ti_3_C_2_T_*x*_−5 [119]. (d) Inactivation performance (i) and EE/O, electrical energy per order (ii) of *E. coli* under UV irradiation and photocatalysis (relative humidity = 95%; initial bacteria concentration: approx. 10^8^ CFU m^−3^; retention time: 4.27 s) [120]. (e) Effect of (i, iii) bacteria species (relative humidity = 30%) and (ii, iv) relative humidity (spore of *B. subtilis*) on inactivation performance and EE/O (retention time: 4.27 s; irradiation intensity: 10 mW cm^−2^) [120].

Antimicrobial synergistic effects between MXenes and metal nanostructures were explored in some of the recent studies, with the aim of developing more effective antibacterial composites based on MXenes. For instance, the AgNPs/Ti_3_C_2_T_*x*_ MXene-based membranes exhibit a significantly higher level of bacterial inhibition in comparison with the pristine polyvinylidene difluoride (PVDF) membrane and Ti_3_C_2_T_*x*_ MXene membrane (figure 5a) [117]. The mechanism of antimicrobial synergism involves the use of MXene nanosheets, which exert physical damage on bacterial cells through their sharp edges. This facilitates the enhanced internalization of AgNPs, leading to the generation of ROS. Consequently, bacterial cell death occurs as a result of oxidative damage to the bacterial membrane and DNA. This antimicrobial effect is observed in both Gram-positive and Gram-negative bacteria [117].

The TiO_2_/Ti_3_C_2_T_*x*_ nanocomposite, while coated onto a polyurethane (PU) foam substrate, showcases a notably superior efficiency in the inactivation of bacteria in air under UV irradiation, as compared with pure TiO_2_ [120]. For wound care applications, the Ti_3_C_2_T_*x*_ (MXene)-incorporated chitosan nanofibres significantly reduce bacterial proliferation (figure 5b) [118]. As Graphene-based materials have also shown appealing photocatalytic properties [22], the MXene-graphene composites are also promising options for developing smart photocatalytic principle-driven sterilizers for combating current and future pandemics. A key factor in enhancing the photocatalytic efficiency of these materials lies in surface functionalization.

The surface functionalization of MXenes has been shown to significantly influence their photoresponse and electrical conductivity [97,121]. Previous studies have demonstrated the remarkable photocatalytic capabilities of carbide MXenes that possess functional groups, such as Ti_2_CO_2_, Zr_2_CO_2_ and Hf_2_CO_2_ [97,121]. The nanocomposite consisting of Ti_3_C_2_ nanosheets and porous g-C_3_N_4_ nanosheets exhibits an interfacial Schottky junction, enabling the reduction of O_2_ in an isopropanol solution to H_2_O_2_ when exposed to visible light irradiation [122]. The recently developed Bi_2_S_3_/Ti_3_C_2_T_*x*_ MXene has also shown enhanced photocatalytic activity, which results in the fast killing of the bacteria [119].

Similarly, a study on the photocatalytic efficiency of a composite consisting of 2D/2D Bi_2_WO_6_/Nb_2_CT_*x*_ has demonstrated the material’s notable efficiency due to the disparity between the conduction band potential of Bi_2_WO_6_ and the Fermi level of Nb_2_CT_*x*_. In this process, electrons undergo migration towards Bi_2_WO_6_ in order to achieve energy band equilibrium, resulting in the formation of a space charge layer and a Schottky barrier. This phenomenon reduces the recombination of charges generated by light exposure, thus enhancing photocatalytic efficiency and ultimately leading to the production of hydroxyl radicals (^•^OH) [123]. The photocatalytic properties of all of these nanocomposites can be harnessed for developing effective antiviral technologies.

### 3.3. Boron nitride

Hexagonal boron nitride (h-BN) has a two-dimensional honeycomb lattice structure wherein nitrogen (N) and boron (B) atoms are organized in an alternating manner. The B–N covalent bonds within this lattice possess a bond length of 1.45 Å, while the interlayer spacings between adjacent boron nitride (BN) layers measure 0.333 nm. These interlayer spacings are maintained by mild van der Waals forces [124,125]. The 2D BN sheet displays distinctive characteristics, including exceptional thermal conductivity, notable chemical stability, significant photocatalytic behaviour and a variety of inherent electrical properties. In comparison with graphene, which possesses a zero band gap, BN nanosheets exhibit characteristics of a wide band-gap semiconductor [126,127]. According to the previous findings, the multilayer h-BN possesses a band gap of 6.07 eV, while the single layer demonstrates a band gap ranging from 5.56 to 5.92 eV. This variation can be attributed to the dispersion of electronic bands resulting from the interaction between different layers [34].

#### 3.3.1. Two-dimensional boron nitride as photocatalysts

BN is classified as an ultrawide band-gap semiconductor. When combined with visible light-responsive photocatalysts, this synergy has proven effective in enhancing photocatalytic activities [128]. Typically, h-BN exhibits photocatalytic properties when exposed to UV radiation. However, through element doping, the photocatalytic range can be extended from the UV region to the visible light spectrum. To date, BN-based materials have showcased significant promise as photocatalysts [129–136].

Based on scavenger results, it is evident that the UV photocatalysis of BN follows a hole-initiated reaction pathway, involving superoxide/hydroperoxyl and hydroxyl radicals. This indicates a co-dependent mechanism where both holes and radical species participate in the process [137,138]. Studies have shown that h-BN is four times more efficient under 254 nm light compared with P25-TiO_2_ under optimal conditions [139]. Additionally, the use of an h-BN UVC photoreactor has demonstrated high efficacy in treating contaminated groundwater [140].

Multiple methods have been proposed by researchers to enhance the light-driven capability of h-BN. These strategies encompass modifying the structural morphology, establishing heterojunctions with compatible photocatalysts and introducing heteroatoms through doping. The presence of diverse structural morphologies leads to the formation of numerous active sites, which in turn facilitate the processes of adsorption and efficient charge transfer [139,141,142].

#### 3.3.2. Antimicrobial application

As of now, the potential of 2D h-BN to exhibit antimicrobial efficacy under light irradiation remains largely unexplored. The several antibacterial studies that were previously done by Gudz *et al.* [143], Ahmed *et al.* [144], Zhang *et al.* [145] and Kivanc *et al.* [146] mainly followed a contact-killing mechanism rather than a photocatalytic approach. Additionally, no comprehensive investigation has been conducted on the antiviral effectivity of boron nitride nanosheets (BNNS).

However, h-BN exhibits photocatalytic properties when exposed to UV radiation. To date, BN-based materials have showcased significant promise in the photocatalytic degradation of various pollutants, including dyes, medicines, heavy metals and organic compounds [129–136]. However, any antiviral study with BNNS has not been reported yet.

#### 3.3.3. Composites and modification

Classified as an ultrawide band-gap semiconductor, BN tends to enhance photocatalytic activities when combined with visible light-responsive photocatalysts [128]. For instance, BN/TiO_2_ nanocomposite shows improved photocatalytic activity under UV-A and UV-C than TiO_2_, while BN remains mostly inactive [136,139,147,148]. Band diagram analysis and photocurrent response measurements suggest that the BN/TiO_2_ heterojunction semiconductor exhibits type-II characteristics, which effectively promotes the separation of charge carriers [147]. The composite is also effective in visible light [147].

Similarly, the use of BNNS enveloped by AgI demonstrates a notably enhanced photocatalytic efficacy in contrast to the pure AgI, when subjected to simulated sunlight. The primary factors contributing to the improved photocatalytic activities are mostly ascribed to the elevated light absorption and separation rate of photo-induced electrons and holes, achieved through the interfacial transfer of photogenerated electrons from the BNNS to the AgI nanostructures [129]. Other composites, such as BN/cadmium aluminate [133], BNNS/CN [149] and BN/bismuth oxybromide [131], also exhibit excellent photocatalytic activity. The modifications to BNNS seem to enhance their photocatalytic capabilities as well. For instance, BN-modified BiPO_4_ demonstrated a noteworthy photocatalytic efficiency under UV light [130], while SnO_2_-modified BN sheets exhibited remarkable potency resulting in substantial hydroxyl radical formation [135].

Metal and non-metal doping in h-BN nanomaterials helps improve their photodegradation performance under visible light irradiation by reducing the band gap of original h-BN [150,151]. The introduction of carbon into the h-BN lattice transforms non-photoresponsive h-BN into a visible light-responsive material [152]. The formation of C–B and C–N bonds significantly alters the electronic environment, resulting in improved visible light activity, reduced charge transfer resistance and increased charge carrier density. This enhancement boosts the photocurrent density compared with pure h-BN. Analysis of the electronic band structure and charge trapping confirms the presence of e^−^, •O_2_^−^ and •OH as the primary ROS [152]. Likewise, the incorporation of transition metals like Cu or Co into BNNS can also enhance the latter’s photocatalytic property and hence improve its antibacterial activity [153–156].

Researchers have explored the antibacterial properties of h-BN-based nanocomposites such as Zr-doped BNNS [157], BN/Ag, BN/Ag-TiO_2_ [158], quaternary ammonium compounds (QACs)/h-BN/linear low-density polyethylene (LLDPE) nanoplatelet [159], functionalized h-BN nanosheets/GO/Ag [160], BN/poly(N-methylpyrrole) [161], etc., against *E. coli*, *S. aureus*, *Pseudomonas aeruginosa* and *Enterococcus faecalis*. Though the composites exhibited significant efficiency in inactivating these bacteria, mechanical force and adhesion-assisted physical damage were the sole reasons behind that. Their photocatalytic property was not studied.

### 3.4. Transition metal dichalcogenides

TMDs are typical 2D layered nanomaterials with three atom layers, forming an ‘X–M–X’ sandwich structure. Their versatile chemistry ranges from metals (e.g. NbS_2_ and VSe_2_), semimetals (e.g. WTe_2_ and TiSe_2_) and semiconductors (e.g. MoS_2_ and WS_2_), to insulators (e.g. HfS_2_). The two common structural phases are characterized by either trigonal prismatic (2H) or octahedral (1T) co-ordination of metal atoms [162–164]. TMDs have a tunable electronic band gap, resulting from the quantum confinement effect, well-placed energy levels and atomically thin nature to give them excellent photocatalytic properties [31,165–168]. Naturally, the band-gap values between different TMD monolayers are also different, which leads to their different spectral responsivities [169]. For instance, when the thickness of MoS_2_ decreases to monolayer, it changes from an indirect band-gap semiconductor to a direct band-gap semiconductor, resulting in a dramatic jump in luminescence [170].

#### 3.4.1. Transition metal dichalcogenides as photocatalysts

Crystal lattices of MoS_2_ exist in four polymorphic forms namely 1H, 2H, 1T and 3R (H—hexagonal, T—tetragonal and R—rhombohedral) which are classified based on the stacking arrangement and the coordination between Mo and S atoms. Among the four polymorphic forms of MoS_2_, 1H–MoS_2_ serves to be the most stable one [171,172]. Atomically thin 2D MoS_2_, featuring an extremely high specific surface area, minimal thickness, powerful quantum confinement of electrons and an optimal band structure, is extensively used in photocatalysis [169,173,174]. Compared with bulk forms, these thin 2D MoS_2_ materials, with their distinctive structure and adjustable electronic characteristics, enhance photocatalytic efficiency through three key processes: light absorption, charge separation and surface catalytic reactions [31]. The few-layered or single-layered 2D MoS_2_ offers more active sites, aiding not only in the absorption of UV-visible light [175] but also in speeding up the transfer of photogenerated electron holes from the semiconductor’s interior to its active surface sites [173].

WS_2_, another well-known TMD, contains two phases, prismatic trigonal 2H and octahedral 1T, that may be changed into each other under certain conditions. Its indirect band gap and greater direct band gap rely on synthesis technique [176]. Researchers have looked into WS_2_’s photocatalytic capability and found few-layered WS_2_ nanosheets to be photocatalytically active under visible light irradiation [177]. The hexagonal-shaped WS_2_ platelets fabricated via hydrothermal route show better photocatalytic activity than the irregular WS_2_ platelets [178]. Furthermore, the exfoliated hexagonal WS_2_ nanosheets exhibit an enhanced photocatalytic activity than h-WS_2_, because of its lower band gap, larger surface area and more active sites [179,180]. Apart from MoS_2_ and WS_2_, TMDs like NbS_2_ [181] and ReS_2_ [182] have demonstrated good photocatalytic behaviour as well.

#### 3.4.2. Antimicrobial application

Researchers have verified that MoS_2_ exhibits strong charge carrier mobility, a wide spectral response range and fascinating optical and physical properties [183]. These findings suggest that MoS_2_ has promising potential for photocatalytic antibacterial applications. Additionally, the size and composition of MoS_2_ significantly impact its antibacterial properties [31,184].

The size and composition of MoS_2_ significantly impact its effectiveness. Studies show that a few-layered MoS_2_ film generates more ROS compared with bulk MoS_2_, entirely inactivating *E. coli* under visible light [31]. The effectiveness of MoS_2_ nanosheets under sunlight depends on both exposure time and concentration. According to the mechanism study, after absorbing the solar light MoS_2_ was excited to singlet MoS_2_, and inter-system crossing rapidly transformed it to triplet MoS_2_, further producing ^•^O_2_^−^, H_2_O_2_ and ^•^OH [184]. In a recent study, the interaction between herpes simplex virus type 1, (HSV-1) with MoS_2_ NS, and GO has been highlighted [18]. However, the mechanism of inactivation is mainly contact killing, which underscores the necessity of harnessing photocatalytic properties.

**Figure 6.**
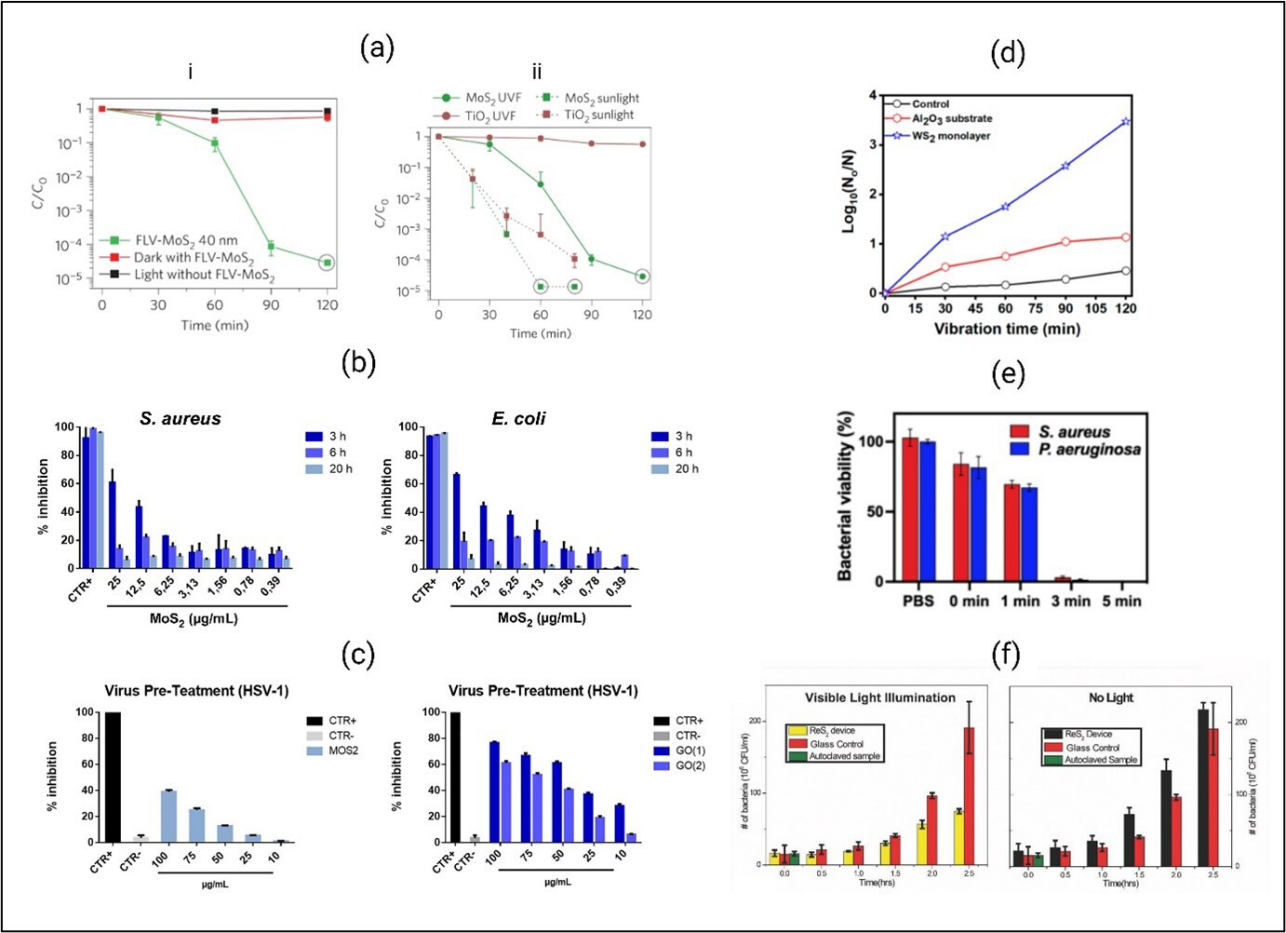
Antimicrobial effectivity of TMDs. (a-i) Comparison of the disinfection performances of few-layered, vertically aligned molybdenum disulfide (FLV-MoS_2_) with both light control without FLV-MoS_2_ and FLV-MoS_2_ in the dark to confirm the visible-light photocatalytic effect. (a-ii) Comparison of disinfection performance between FLV-MoS_2_ and TiO_2_ films under visible-light and real-sunlight illumination in the disinfection performances [31]. (b) Percentage of bacterial growth inhibition after treatment with various concentrations of MoS_2_ NSs. Ampicillin and vancomycin represented the positive controls for *E. coli* and *S. aureus*, respectively. The negative controls consisted of deionized water. The data statistical error is the standard deviation of three independent experiments. *p* < 0.05 [18]. (c) HSV-1 infectivity inhibition with MoS_2_ and GO NSs. Not infected and not treated cells represented the positive control (CTR+), meanwhile infected cells were the negative control (CTR−). The data statistical error is the standard deviation of three independent experiments. *p* < 0.05 [18]. (d) Log reduction of CFUs ml^−1^ of *E. coli* with time when treated with 2D WS_2_ monolayer [185]. (e) Bacterial viability of *S. aureus* and *P. aeruginosa* treated by NbS_2_ NSs *in vitro* under different times (0−5 min) of 808 nm laser (2.0 W cm^−2^) on tryptic soy broth (TSB)-agar plates [181]. (f) *Escherichia coli* disinfection on exposure to visible light and dark/no light using vertically oriented ReS_2_ nanosheets. The experiments were run for approximately 2.5 h in TSB growth media [186].

In addition to MS_2_, other TMDs such as WS_2_, NbS_2_, RS_2_ and so on, have exhibited noteworthy photocatalytic activity. A uniform WS_2_ monolayer shows its excellent photocatalytic performance by eradicating almost 100% of *E. coli* within 2 h [185]. Similarly, the exfoliated hexagonal WS_2_ displays an enhanced photocatalytic activity compared with h-WS_2_, because of the former’s lower band gap, larger surface area and more active sites [179]. The 2D NbS_2_ nanosheets eliminate *P. aeruginosa* and *S. aureus* under 5 min of 808 nm laser irradiation (figure 6e) [181]. Moreover, when exposed to NIR radiation, NbS_2_ nanosheets not only prevent the formation of biofilms but also rapidly eliminate *S. aureus* bacteria from an infected lesion, thereby facilitating wound healing by inducing hyperthermia. Ghoshal *et al.* demonstrated the significant potential of vertically oriented ReS_2_ nanosheets for photocatalytic water disinfection (figure 6f) [186]. Nevertheless, the effectiveness of the inactivation of the system still requires additional enhancement because to the swift recombination of the carriers.

#### 3.4.3. Composites and modifications

Several MoS_2_-based composites have demonstrated potent antibacterial activity through photocatalytic ROS generation. For instance, the ultra-thin MoS_2_/α-NiMoO_4_ type II heterojunction inactivates *S. aureus* in water within 150 min through the formation of ROS [187]. Likewise, the Z-scheme MoS_2_/Ag_2_CO_3_ composite completely inactivates *E. coli* under visible light irradiation and exhibits better photocatalytic activity than MoS_2_ and Ag_2_CO_3_ alone [188]. The presence of Cu^2+^ in the CuNPs/MoS_2_ composite facilitates the separation of e^−^/h^+^ pairs, increasing the yield of ROS in MoS_2_ nanosheets. Notably, the composite demonstrates greater antibacterial performances towards *E. coli* and *S. aureus* than Cu or MoS_2_, and the bactericidal effect lasts for at least 6 h [189]. Silver incorporation, as seen in the Ag/MoS_2_-Ti heterojunction, also leads to faster charge transfer and rapid disinfection of *S. aureus* and *E. coli* under visible light [190].

**Figure 7.**
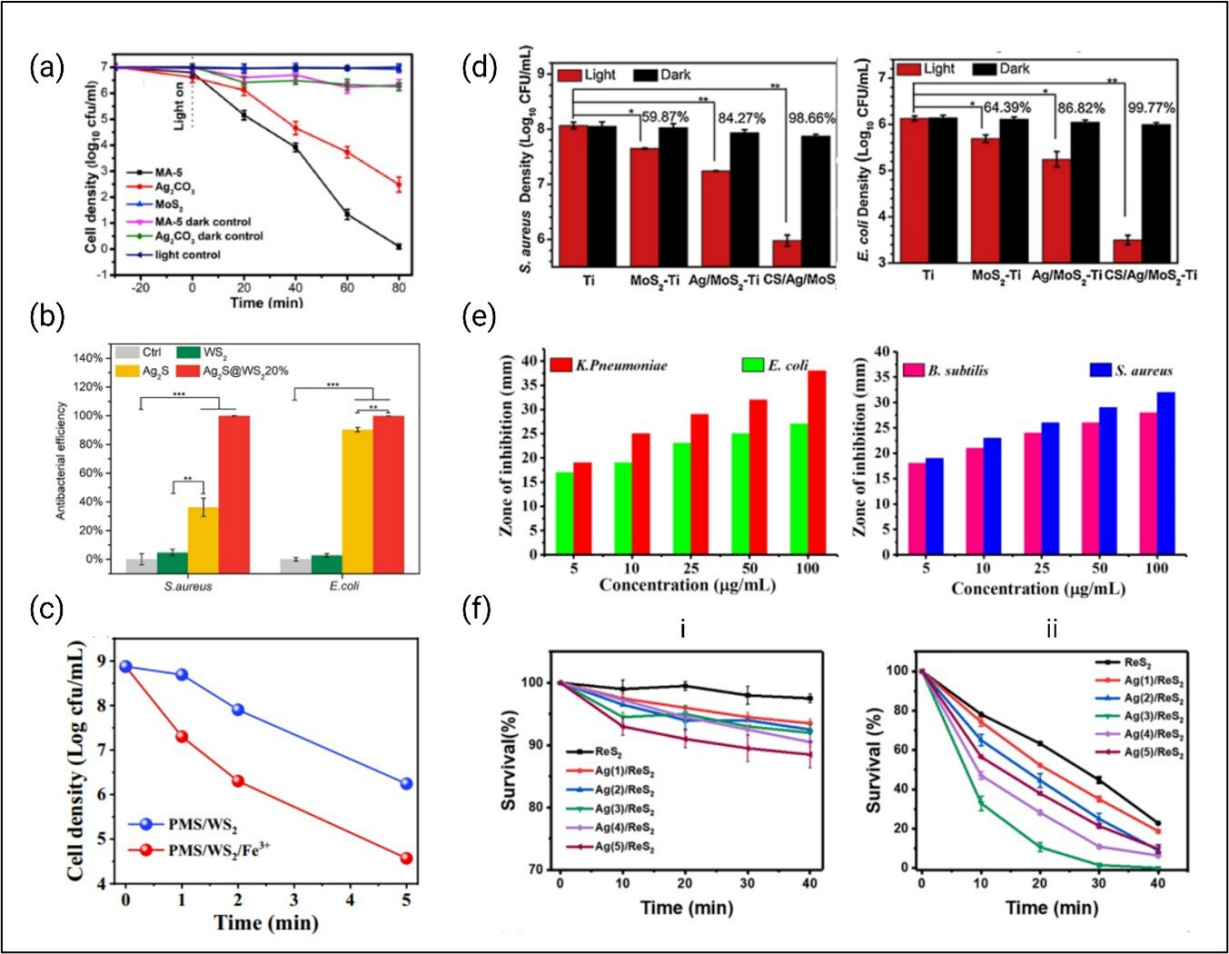
Photocatalytic disinfection property of TMD-based composites. (a) Photocatalytic inactivation efficiency of *E. coli* in the presence of the MoS_2_/Ag_2_CO_3_ composites under visible light [188]. (b) Antibacterial capability of WS_2_-based composite against *S. aureus* and *E. coli* after 20 min irradiation (*n* = 3, mean ± s.d.: *p < 0.05, **p < 0.01, ***p < 0.001) [191]. (c) Inactivation of *E. coli* in different WS_2_-based reaction systems (pH = 4.0, [WS2] = 0.4 g l^−1^, [Fe^3+^] = 0.1 mM, [PMS] = 0.15 mM, [*E. coli*] = 1 × 10^8^ CFU ml^−1^) [192]. (d) Comparison of the antibacterial activities among various MoS_2_-based composites against *S. aureus* and *E. coli* after irradiation by 660 nm light or staying in dark for 20 min. The error bars indicate means ± s.d., *n* = 3. *p < 0.05, **p < 0.01, ***p < 0.001 [190]. (e) Zone of inhibition of CS/WS_2_/Pd composite against *Klebsiella Pneumoniae*, *E. coli*, *B. subtilis*, *S. aureus* [193]. (f) Photocatalytic sterilization efficiency of Ag/ReS_2_ composite with different Ag content grown on pyridinium chlorochromate (PCC) substrate, against *E. coli* (10^4^ CFU ml^−1^) under dark condition (i) and visible light (ii) [194].

Similar to MoS_2_, tungsten disulfide (WS_2_) benefits from the elemental modifications. The Ag_2_S/WS_2_ composite showcases an enhanced photocatalytic efficiency under NIR light irradiation against *S. aureus* and *E. coli*, with almost 100% eradication within 20 min (figure 7b) [191]. The material is also non-cytotoxic, indicating its potential for rapid and effective disinfection. The incorporation of silver nanoparticles in the WS_2_ monolayer significantly increases the antibacterial activity of the latter [195]. Researchers have also reported an enhanced disinfection of *E. coli* after introducing Fe^3+^ into 2D WS_2_ [192]. The antibacterial efficiency of the chitosan/WS_2_/Pd composite has been investigated against *B. subtilis*, *S. aureus*, *Klebsiella pneumoniae* and *E. coli* [193]. The liquid-phase-exfoliated bioactive CS/WS_2_/Ru composite demonstrates similar bacterial inhibition [193]. Apart from these, WS_2_/g-C_3_N_4_ [196], WS_2_/MoS_2_ [197], WO_3_/WS_2_/polyaniline (PANI) [198], ZnO/WS_2_ [199], WS_2_/TiO_2_ [200], shows good photocatalytic activity.

Rhenium disulfide (ReS_2_) composites offer additional capabilities. The Ag/ReS_2_ heterostructure enables complete inactivation of *E. coli* within 30 min under visible light (figure 7f) [194]. The presence of Ag nanoparticles on the surface of ReS_2_ nanosheets serves two purposes. Firstly, they function as electron capture agents, facilitating the separation of photogenerated carriers. Secondly, they suppress the recombination of photogenerated carriers. Notably, the size of Ag NPs has a pivotal role in this phenomenon [201]. Further, the BaTiO_3_/ReS_2_ piezo-assisted photocatalysis heterostructure developed has proven its high degradation efficiency [202].

Beyond MoS_2_, WS_2_ and ReS_2_, other 2D materials have been explored. For example, the NiS_2_/g-C_3_N_4_ composite exhibits enhanced photocatalytic efficiency due to intimate nanosheet contact between NiS_2_ and g-C_3_N_4_, which facilitates charge transfer and suppresses recombination [203].

### 3.5. Titanium dioxide

Titanium dioxide (TiO_2_) is an oxide semiconductor photocatalyst that is found in three main crystalline structures: rutile, anatase and brookite. Rutile and anatase are the predominant types used, with rutile exhibiting greater stability. Atomic-level thin TiO_2_ nanostructures in a 2D form possess unusual features such as a high surface-to-volume ratio, unique electrical properties and interesting chemical reactivity. The ultrathin 2D TiO_2_ nanosheets exhibit exceptional photochemical properties due to their distinctive characteristics such as single-crystallinity, atomic-level thickness and highly reactive exposed surfaces [204,205].

#### 3.5.1. Two-dimensional titanium dioxide as photocatalysts

Being a semiconductor, TiO_2_ comprises a small energy difference between the conduction and valence bands. Electrons that are present in the valence band get excited to the conduction band when light (UV) falls on its surface. This phenomenon leads to the formation of both negative and positive charges on the semiconductor surface. Titanium dioxide has a band gap (3−3.2 eV) with a maximum absorption wavelength of 400 nm [206]. The predominant reactive species upon UV exposure is hydroxyl radical and the efficiency is highly dependent on water matrix properties, such as pH, and organic/inorganic species [207].

#### 3.5.2. Antimicrobial applications

Among the photocatalysts employed, TiO_2_ is one of the most extensively used photocatalysts for antimicrobial applications [206]. So far, the effectiveness of photocatalytic nanomaterials, including TiO_2_-based nanostructures, in disinfection of a range of viruses, such as human adenovirus GB, influenza A and B, hepatitis B, avian influenza virus (A/H_5_N_2_) and norovirus, has been reported, thus indicating the great promise of this class of materials in the fight against the risks deriving from these pathogens [208,209].

Recent studies have highlighted the effectiveness of TiO_2_ in disinfecting the SARS-CoV-2 virion in both air and liquid [210]. TiO_2_-mediated photocatalytic reactions reduce viral infectivity by about 99% in aerosols within 20 min, while achieving the same reduction in liquids takes 120 min. This disinfection occurs due to damage to the viral proteins and genome through the mechanistic effects [210]. Additionally, TiO_2_ has been shown to induce membrane rupture, emphasizing its potential in antimicrobial applications [211].

**Figure 8.**
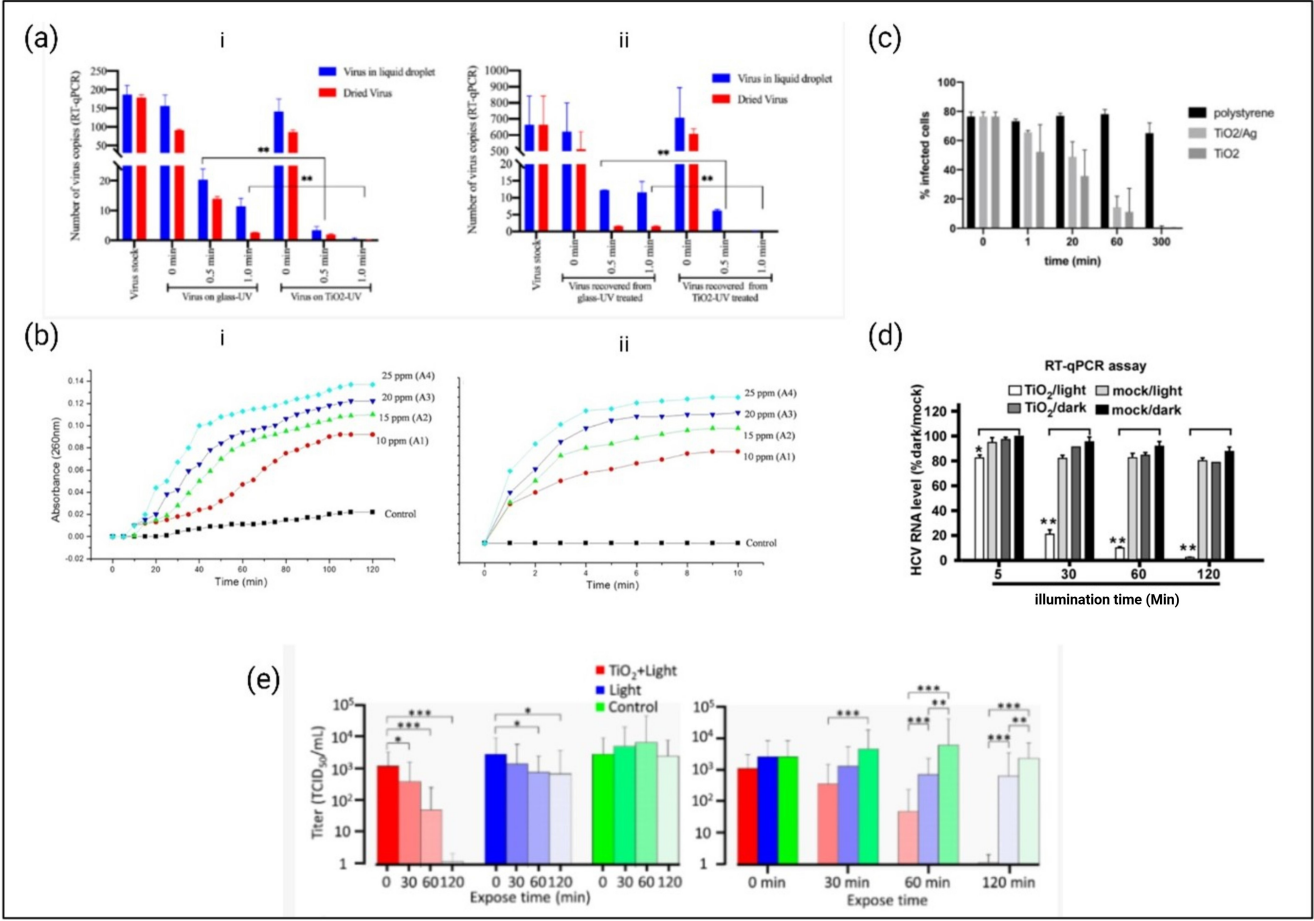
Antimicrobial performance of TiO_2_. (a) trinitrophenol (TNP)-coated surfaces inactivate wet and dried HCoV-NL63. Virus was deposited on TiO_2_-treated and untreated coverslips, then exposed to UV light either immediately or after drying (approx. 20 min). Virus recovery was performed using phosphate buffer saline. (i) Viral genome quantification via reverse transcription quantitative polymerase chain reaction (RT-qPCR). (ii) Infectious virus quantified in HEK293L cells after 48 h infection. Statistical analysis: two-way ANOVA, **p < 0.01 [213]. (b) (i) Release of 260 nm absorbing material from *E. coli* treated with varying nano-titania concentrations, indicating membrane damage. (ii) Uptake of 1-N-phenylnaphthylamine (fluorescence intensity) by *E. coli*, reflecting membrane damage and dysfunction. A1–A4: nano-titania concentrations (10–25 ppm) [211]. (c) Effect of TiO_2_/Ag and TiO_2_-coated tiles on. SARS-CoV-2 isolate REMRQ0001/Human/2020/Liverpool [212]. (d) Photo-activated TiO_2_ inhibits hepatitis C virus (HCV) infection. 1000 ffu HCV (JFH1 strain) was treated with TiO_2_ ± light for 5, 30, 60 and 120 min, then inoculated into Huh7.5.1 cells for 3 days. HCV RNA levels were measured by RT-qPCR, normalized to glyceraldehyde-3-phosphate dehydrogenase (GAPDH), and expressed as a percentage of the mock/dark group [214]. (e) Inactivation of SARS-CoV-2 in liquid by LED-TiO_2_ photocatalytic reaction. After the photocatalytic reaction, viral titre was confirmed by TCID50 assay. (*p < 0.05; **p < 0.01; ***p < 0.001) [210].

While interacting with two different strains of SARS-CoV-2, spike pseudo-typed virions and fully infectious virus, photocatalysis of TiO_2_ leads to a free radical attack on viruses and results in four orders of magnitude decrease in spike viral load after 1 h, with no active virus detected after 5 h [212]. The TiO_2_ P25-based coatings effectively inactivate human coronavirus (HCoV-NL63) in both wet and dry environments under UV light exposure (figure 8a) [213]. In addition, the SARS-CoV-2 pseudo-virus can be adsorbed on the TiO_2_ surface, followed by ROS attack [215]. Regarding a broad range of pathogens including SAR-CoV-2 and Hepatitis C, nanosized TiO_2_ attacks the viral ribonucleic acid genome through hydroxyl groups and inhibits the pathogens under low irradiation (figure 8d) [214]. Other studies suggest the generation of titanium radicals that damage proteins, DNA and lipids, with minimal cytotoxic effect on the cellular host [216]. Seven minutes of UV irradiation on TiO_2_ coating entirely eliminated influenza virus A/PR/8/1934 (H1N1) [217] and MS2 bacteriophage [218]. Moreover, a titania-based indoor UV air disinfector has been developed that shows complete removal of *B. subtilis* spores within an hour [219].

Similarly, a significant reduction in viral titre, ranging from 2.4 to 2.8 logs, has been found while applied to a bovine coronavirus suspension and exposed to visible light [220]. The inactivation mechanism is hypothesized to involve the formation of a peroxotitanium complex, which induces physical damage, metal ion toxicity and photocatalytic oxidation facilitated by photogenerated ROS [220]. The efficacy of such TiO_2_ coating under typical indoor light conditions makes it a promising option for surface disinfection.

#### 3.5.3. Composites and modifications

Introducing chalcogens (S, Se, Te, etc.) significantly enhances the antimicrobial properties of TiO_2_ [221]. For instance, Te-doped TiO_2_ displays the highest efficacy against *E. coli*, achieving complete disinfection in just 70 min under light exposure. Similarly, Ce-doped TiO_2_, applied on reduced graphene oxide (rGO) exhibits improved activity under visible light. The improvement is ascribed to cerium’s capacity to expand titanium’s light absorption range from UV to visible spectrum [222]. TiO_2_-based nanocomposites, such as TiO_2_/chitosan [223], iron-doped TiO_2_ [224], LaFeO_3_/TiO_2_ [225], Fe_3_O_4_-TiO_2_/GO [226] demonstrate photoactivity under visible light at different pH conditions.

In a recent study, a TiO_2_-reduced GO composite successfully inactivated even antibiotic-resistant *E. coli* and *P. aeruginosa* in real urban wastewater within 3 h [227]. This opens doors for tackling increasingly challenging microbial threats. Silver (Ag)-incorporated composites such as GO/TiO_2_/Ag further enhance this effect, demonstrating a greater decrease in bacterial viability after just 10 min of solar treatment [228]. Additionally, Cu/TiO_2_ coatings synthesized through a sol–gel process showcase efficient disinfection upon prolonged exposure to UVA light [229].

**Figure 9.**
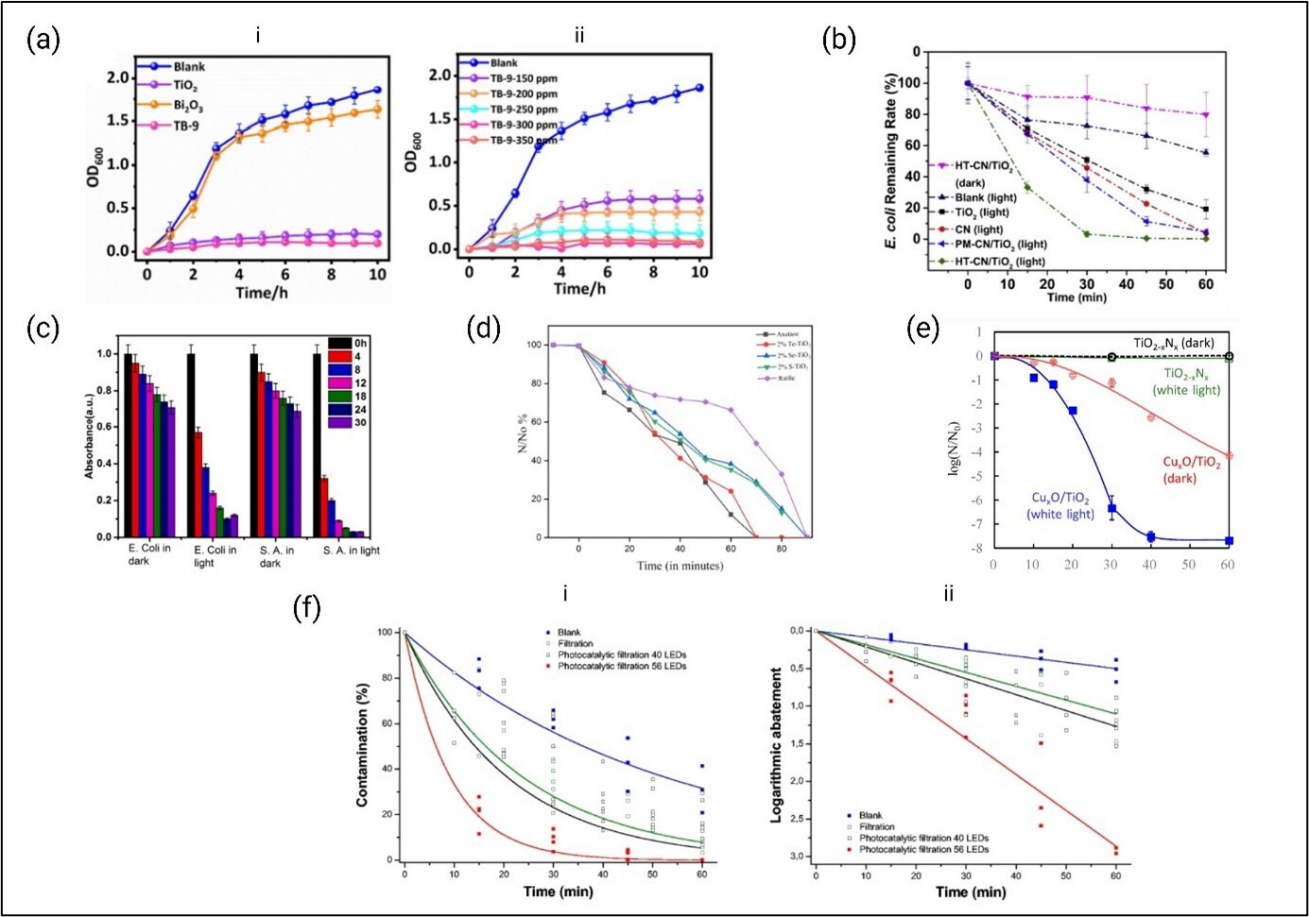
Antimicrobial activity of TiO_2_-based composites. (a-i) Growth curves of *E. coli* over TiO_2_, Bi_2_O_3_ and TB-9 (TiO_2_/Bi_2_O_3_); (a-ii) growth curves of *E. coli* using different concentrations of the TB-9 sample. Each experiment was repeated four times [230]. (b) Inactivation performance of the photocatalysts in simulated solar light towards *E. coli* and control groups in the dark and in the absence of photocatalyst [231]. (c) Cell growth inhibition of bacterial cells when treated with 0.2%Ce-TiO_2_@10%rGO in dark and under visible light [222]. (d) Photocatalytic inactivation of *E. coli* with anatase, rutile, chalcogen (S, Se and Te doped) TiO_2_ [221]. (e) Inactivation of bacteriophage Qβ: Cu_*x*_O/TiO_2_ under white light (blue) and dark (red); TiO_2_-xNx under white light (green) and dark (black). Light exposure used a 10W fluorescent bulb with UV cut-off, 800 lux [232]. (f) Decontamination kinetics of T2 bacteriophage bioaerosol using 40 and 56 LED TiO_2_/β-SiC foam photocatalytic reactors, showing (i) contamination over time and (ii) logarithmic reduction [233].

A vertical junction of TiO_2_ nanosheets and graphitic carbon nitride rapidly disinfects *E. coli* (log 3 in 30 min under sunlight) due to its large surface area and efficient Z-scheme charge transfer (figure 9b). This nanocomposite outperforms pure materials by promoting ROS generation for enhanced bacterial inactivation [231]. On the other hand, the 2D TiO_2_/Bi_2_O_3_ nanosheet composite uses an S-scheme mechanism to efficiently separate charges that boosts ROS production and inactivates *E. coli* (4.63 × 10^7^ CFU ml^−1^ in 6 h) because of its close 2D interface and extended charge carrier life (figure 9a) [230].

The impact of TiO_2_ nanocomposites extends even further, targeting viruses as well. The CuO/TiO_2_ composite effectively reduces viral contamination on surfaces and inactivates Qb bacteriophage when exposed to aerosolized viral suspension [232]. Similarly, TiO_2_/hydroxyapatite (HA) composite has been developed for the purpose of an antiviral filtration system [234]. The material uses the photocatalytic behaviour of TiO_2_ nanoparticles under UV light, along with the adsorption capabilities of hydroxyapatite, to eliminate the H_1_N_1_ influenza A virus [234]. Furthermore, the use of TiO_2_/b-SiC for air purification from airborne T2 bacteriophage under 1 h UV exposure has achieved almost 3 log reduction (figure 9f) [233].

Beyond the previously mentioned materials, TiO_2_ nanosheets combined with layered BP maintain a high photocatalytic performance under both UV and visible light, retaining 92.5% efficiency even after three cycles. This is the result of improved light absorption and reduces electron–hole pair recombination facilitated by the composite’s formation [235]. Similarly, the 2D TiO_2_/g-C_3_N_4_ nanosheet heterojunctions have exhibited notable photocatalytic performance under simulated solar light compared with pure g-C_3_N_4_ and TiO_2_ nanosheets [236]. The material’s enhanced photocatalytic activity is attributed to a direct Z-scheme charge transfer mechanism, where superoxide radicals (•O^2–^) and photoiinduced holes (h^+^) are the primary active species responsible [236].

### 3.6. Other nanomaterials

Composites based on graphitic carbon nitride have demonstrated commendable photocatalytic performance so far. Notably, porous g-C_3_N_4_ (CN) incorporated with black phosphorus nanosheets (BPNS) shows enhanced photodegradation efficiency compared with the pristine CN [237]. The dual heterojunction photocatalyst, consisting of p-n and Z-scheme interfaces, facilitates the directed migration and efficient separation of photogenerated carriers. Additionally, it maintains a greater redox potential, enabling the simultaneous generation of O_2_ radicals and OH^−^ radicals [237].

A ternary nanocomposite consisting of graphitic carbon nitride (g-C_3_N_4_), rGO and iron oxide has been explored under visible light in the presence of hydrogen peroxide. The nanocomposite has been found to disrupt supercoiled DNA, transforming it into single-stranded circles before fragmenting it further [238]. The increased efficiency can be attributed to rGO’s facilitation of charge carrier relaxation, g-C_3_N_4_’s heightened activity, ROS production from H_2_O_2_ and iron oxide’s photo-Fenton reactions [238]. Researchers have also developed an Ag/AgBr/g-C_3_N_4_ composite for the inactivation of tetracycline-resistant *E. coli* [239]. Separate studies have shown g-C_3_N_4_’s effectiveness against multidrug-resistant Enterobacteriaceae, with its visible light photoactivity and low cytotoxicity [240].

Apart from g-C_3_N_4_, cadmium sulfate (CdS) nanoparticles have emerged as a remarkable visible-light-driven photocatalyst with impressive antibacterial properties [241]. Moreover, the innovative combination of tungsten trioxide-based photocatalysis with copper nanoclusters has proven to be highly effective in deactivating the SARS-CoV-2 virus in indoor conditions [242]. These cutting-edge photocatalysts offer immense potential for applying antimicrobial coatings to surfaces, making them highly suitable for both outdoor and indoor environments [52].

Table 1 provides a comprehensive insight on the structural, functional and antiviral properties of the 2D materials discussed so far.

**Table 1.** Comparison of structural, functional and antiviral properties of prominent 2D nanomaterials.

2D material	essential properties	antibacterial and antiviral activity	functionalization and composites	references
black phosphorus (BP)	band gap: 0.3−2.0 eV; biodegradable; UV-visible-NIR absorption	kills *E. coli*, *S. aureus*; 99.3% efficiency in 10 min (visible light); layer-dependent	BP/TiO_2_, BP/Ag, polymeric coatings; enhanced ROS, stability	[33,54–81]
MXenes	formula M_*n*+1_X_*n*_T_*x*_; e.g. Ti_3_C_2_T_*x*_, Zr_2_CO_2_; broad UV–IR absorption	effective against *E. coli*, MRSA, SARS-CoV-2; size, stoichiometry-dependent	AgNPs, TiO_2_ composites, chitosan; graphene hybrids	[35,82–85,91–123]
boron nitride (BN)	h-BN: band gap 5.56−6.07 eV; chemically stable; thermally conductive	primarily antibacterial via contact; limited/no antiviral study	BN/TiO_2_, BN/AgI, BN/metal doped; active under UV/visible	[34,124–127,129–160]
transition metal dichalcogenides (TMDs)	e.g. MoS_2_, WS_2_, ReS_2_; layer-dependent band gap; high surface area	*E. coli*, *S. aureus*, HSV-1; near 100% inactivation (WS2, 2 h)	MoS_2_/Ag_2_CO_3_, WS_2_/Fe3+, ReS_2_/Ag; high ROS generation	[31,162–203]
graphitic carbon nitride (g-C_3_N_4_)	band gap ~ 2.7 eV; visible-light active; polymeric	multidrug-resistant *E. coli*, Enterobacteriaceae; efficient ROS	g-C_3_N_4_/BP, rGO/FeO_*x*_, Ag/AgBr/g-C_3_N_4_	[237–240]
titanium dioxide (TiO_2_)	band gap: 3−3.2 eV; UV-active; anatase/rutile forms	HCoV-NL63, SARS-CoV-2, H1N1, *E. coli*; up to 99% in 20 min	Ag, Cu, Ce-doping, TiO_2_/g-C_3_N_4_, TiO_2_/Bi_2_O_3_	[204–236]

## 4. Evaluation of photocatalytic antiviral efficacy: model viruses, standard assays and key considerations

As research on emerging 2D nanomaterials for antiviral applications expands, rigorous evaluation of antiviral efficacy becomes crucial for comparing and validating their performance. This section examines how the antiviral activity of photocatalytic 2D nanomaterials is assessed, highlighting the common viral strains used as model pathogens and the standard assays employed to measure virus inactivation. Notably, the susceptibility of viruses can vary depending on their structure. For instance, enveloped viruses often show different inactivation profiles compared with non-enveloped viruses, which underscores the importance of selecting appropriate viral models for testing [243]. Furthermore, recognizing these factors and the broader experimental challenges is vital for interpreting results and refining the design of nanomaterials for enhanced antiviral efficacy in real-world applications.

### 4.1. Common viral strains used in antiviral studies

Research on antiviral nanomaterials commonly evaluates both enveloped and non-enveloped viruses to gauge broad-spectrum efficacy. Enveloped viruses frequently tested include influenza A viruses (e.g. H1N1 and H3N2 strains) and coronaviruses [244]. Studies have examined human respiratory coronaviruses such as HCoV-229E and SARS-CoV-2, as well as animal coronaviruses like transmissible gastroenteritis virus (TGEV) and porcine epidemic diarrhoea virus (PEDV) as surrogates. Other enveloped viruses used include poxviruses (e.g. vaccinia virus strain MVA) and feline coronavirus (FCoV), the latter often serving as a model for human coronaviruses [244,245]. For non-enveloped viruses, researchers commonly employ robust enteric virus surrogates such as feline calicivirus (FCV) or murine norovirus (MNV) to represent human norovirus. Adenovirus (a non-enveloped DNA virus) and small enteroviruses like poliovirus and coxsackievirus are also included to test resistance of naked viruses. Bacteriophages, e.g. MS2 (non-enveloped) or Phi6 (enveloped) are occasionally used as well to model virus behaviour under various conditions [243–245]. Notably, international standard tests mandate using one enveloped and one non-enveloped virus. For example, ISO 21702:2019 (antiviral activity on plastics) specifies testing with an influenza A H3N2 strain and feline calicivirus as representatives of enveloped and non-enveloped viruses, respectively. This ensures that new 2D nanomaterial-based antivirals are evaluated against both virus types, such as an influenza virus (enveloped) and a norovirus surrogate (non-enveloped), to demonstrate broad antiviral potential.

### 4.2. Standard methods for assessing antiviral effectiveness

Evaluating antiviral efficacy relies on virological assays that quantify infectious virus remaining after treatment. The plaque assay is a gold-standard method for measuring viable virus titre: samples are applied to a monolayer of host cells and the number of plaques (zones of infected cell lysis) is counted, yielding a titre in plaque-forming units (PFU) [246,247]. Similarly, the 50% tissue culture infectious dose (TCID50) assay is widely used, in which serial dilutions of a virus are applied to cells to determine the dilution that infects 50% of cultures; results are typically reported in TCID50 per ml [248,249]. These infectivity assays are considered definitive for antiviral tests, as they reflect the reduction in live infectious virus after exposure to the nanomaterial under illumination. For instance, in photocatalytic surface studies following ISO 21702, a known virus inoculum is placed on the material, incubated (often 1−24 h), then recovered and titrated by plaque assay or TCID50 to calculate log10 reduction in viral titre. The antiviral activity is expressed as a log reduction (*R*), e.g. *R* = log10(virus_titre_control) – log10(virus_titre_sample) [250].

Molecular methods complement these infectivity assays. Real-time RT-qPCR is commonly employed to quantify viral RNA genomes remaining after treatment [246,251]. While PCR is sensitive and useful for tracking viral load, it detects both intact and inactivated virus particles. Thus, a treated sample might show minimal infectious virus by plaque assay yet still yield high genome copy numbers by RT-qPCR. For this reason, RT-qPCR data are often interpreted alongside infectivity data [246,252,253]. A decrease in PCR signal indicates genome damage or removal, whereas persistence of RNA with loss of infectivity suggests the nanomaterial may have damaged the virus in a way that prevents replication without fully degrading the RNA [243,250,254]. In photocatalysis studies, researchers often use a combination of assays: plaque or TCID50 to measure virucidal efficacy, and RT-qPCR to confirm reduction in viral gene copies. Other techniques like virus plaque reduction assays (testing the material’s ability to inhibit plaque formation) or immunostaining methods can also be employed, but plaque assays remain the gold standard for quantifying antiviral performance of surfaces and materials [250,254].

To ensure consistency and relevance, experiments follow standardized protocols where available. In addition to ISO 21702 for non-porous surfaces, ISO 18184:2019 provides a standard method to evaluate antiviral activity on textiles. These standards specify details like virus inoculum volume, contact time, humidity and recovery media to improve reproducibility across studies. Analogous ASTM standards exist as well (for example, ASTM E1052 for viruses in suspension and E1053 for dried surface disinfectant tests), which many researchers adapt for nanomaterial antiviral testing. Following such protocols, including appropriate controls (virus on an untreated surface, dark conditions versus light activation) and use of neutralizing agents to quench any residual disinfectant activity when recovering the virus, is critical for obtaining reliable, comparable results. For instance, ISO-guided tests incubate virus on surfaces at controlled temperature and humidity (often 25°C, approx. 90% RH for 24 h) and use a neutralizing broth to stop further viral inactivation before titration. Adhering to these methodologies allows researchers to rigorously assess how well a 2D photocatalytic nanomaterial inactivates viruses under defined conditions.

### 4.3. Enveloped versus non-enveloped viruses: differential responses

A consistent finding in antiviral studies is that enveloped viruses are generally more susceptible to photocatalytic inactivation as compared with non-enveloped viruses. Enveloped viruses (such as influenza A and coronaviruses) possess a lipid bilayer envelope that is relatively fragile. ROS generated by photocatalytic nanomaterials can peroxidize and disrupt this lipid membrane and its embedded proteins, leading to rapid loss of infectivity. By contrast, non-enveloped viruses (e.g. norovirus, adenovirus, poliovirus) are protected only by a robust protein capsid, which tends to be more resistant to chemical and physical stress. As a result, non-enveloped viruses often require longer exposure or more aggressive conditions to achieve the same level of inactivation [243,255]. For example, Nakano *et al.* observed that a non-enveloped calicivirus needed roughly twice the UV/TiO_2_ exposure time to be inactivated compared with an enveloped influenza virus [256]. The authors concluded that damage to the lipid envelope of influenza facilitated more rapid structural degradation to underlying capsid proteins and genome than in the sturdier naked virus, which lacks a lipid layer. This is supported by broad-spectrum tests. One study noted TiO_2_ photocatalysis inactivated influenza (enveloped) significantly faster than feline calicivirus (non-enveloped) under identical conditions [243,255]. Similarly, antiviral polymer coatings have shown stronger effects on enveloped viruses like influenza A or vesicular stomatitis virus, whereas tough non-enveloped viruses (e.g. human adenovirus 5) often show reduced or no susceptibility under the same treatment. Non-enveloped viruses are known to be more stable on surfaces and resistant to disinfectants. They survive harsher conditions and are less affected by detergents [250,254]. This explains the greater challenge they pose. Effective antiviral 2D nanomaterials therefore must be validated against non-enveloped viruses to ensure they can tackle those more resistant pathogens.

### 4.4. Challenges in evaluating photocatalytic antiviral efficacy

While many 2D nanomaterials show promise as antiviral agents, accurately evaluating their efficacy comes with several challenges. One major issue is reproducibility and standardization of antiviral tests. Results can vary depending on the virus strain, assay type and experimental set-up. For instance, the innate biological variability between viruses, even between strains of the same virus, can lead to different outcomes as seen in studies where SARS-CoV-2 variant strains showed differing ratios of infectious titre to RNA copies [257]. This means protocols must be carefully standardized by using the same reference viruses, inoculum levels, contact times and light irradiance to compare results across studies. The introduction of standards like ISO 21702 and 18184 has improved consistency, but many published studies still use custom methods, making direct comparison difficult. Achieving statistical confidence in antiviral performance requires multiple independent experiments, and slight changes (e.g. in humidity or organic soiling on the test surface) may impact the observed antiviral effect.

Another challenge is ensuring that the measured antiviral effect is due to photocatalytic nanomaterial and not experimental artefacts. Photocatalytic materials require light activation, so testing must distinguish dark inactivation (if any) from light-driven effects. Control experiments in the absence of light are essential. Similarly, some nanomaterials (or their leached ions) can be cytotoxic to the mammalian cells used in plaque assays, which can confound results by killing the indicator cells rather than the virus [258,259]. To address this, researchers often dilute or neutralize the samples after virus exposure but before titration. Choosing an appropriate neutralizing agent that quenches residual ROS or metal ions without harming the virus or cells is non-trivial but critical for reliable assays. If residual photocatalytic activity continues during the assay, it could falsely exaggerate hypothesized antiviral efficacy.

Lastly, bridging the gap between laboratory tests and real-world efficacy remains challenging. Laboratory antiviral assays typically use controlled volumes of virus in clean suspensions, whereas actual contaminated surfaces may have drying bodily fluids or organic residues that can impede photocatalytic action. Besides, organic matter can consume generated ROS [260]. The intensity and spectrum of light available in real settings (e.g. indoor lighting) might be much lower than the idealized conditions used in experiments, which sometimes employ UV lamps or high-intensity light to activate the catalyst. Thus, a material that inactivates a virus in 30 min under strong UV might perform differently under dimmer ambient light. These factors underscore the importance of simulating practical conditions when evaluating antiviral 2D nanomaterials.

## 5. Practical applications and safety of two-dimensional nanomaterials

### 5.1. Real-world implementations

Two-dimensional photocatalytic materials have begun to translate into real-world antiviral technologies across air, surface and water domains. In air filtration systems, photocatalyst-coated filters and purifiers are used to deactivate airborne viruses. For example, TiO_2_-based filters combined with UV or solar irradiation have achieved significant viral removal in aerosols (up to approx. 99% virus reduction in optimized set-ups) [52,233]. Emerging visible-light-responsive 2D materials such as g-C_3_N_4_ and TiO_2_/g-C_3_N_4_ composites offer the advantage of using indoor light. These materials have demonstrated complete inactivation of model viruses like MS2 bacteriophage in water under visible light (8-log PFU reduction in 240 min), highlighting their potential for air and heating, ventilation, and air conditioning (HVAC) disinfection as well [261]. MXenes and graphene-based materials are also being explored for air purification, not only as passive filters but as active photocatalytic agents. For instance, MXene/graphene composite coatings on air filters could synergistically trap and inactivate viruses under illumination, leveraging MXenes’ light-to-heat conversion and graphene’s high surface area [262].

In surface coatings, 2D photocatalysts are applied to frequently touched surfaces or fabrics to create self-disinfecting materials. Standard antimicrobial coatings (e.g. TiO_2_ on hospital tiles or glass) can inactivate viruses upon UV exposure, Newer 2D formulations aim to work under ambient light. A visible-light-active TiO_2_ nanocoating (peroxo-modified anatase) was shown to reduce a bovine coronavirus titre by approximately 2.5 log10 (around 99.7% reduction) in 4 h under indoor lighting [52]. Similarly, BP nanosheets and semiconducting TMDs like MoS_2_ have been formulated into sprayable surface coatings that generate ROS under sunlight, effectively disinfecting viruses and bacteria on contact [31,75,263,264]. Graphene-based photocatalytic paints and films have been reported as well. For example, composites of graphene oxide with light-sensitive semiconductors can be applied to door handles or countertops to continuously destroy viral particles under room light [25,265]. These surface implementations emphasize the practical benefit of 2D materials’ high surface-to-volume ratios: even thin coatings can provide a dense layer of photocatalytic sites for virus adsorption and inactivation.

In water purification, 2D photocatalysts have shown strong antiviral performance in treating virus-contaminated water using solar illumination. Graphene-based materials and g-C_3_N_4_ are attractive here because they avoid the use of UV lamps and can function with sunlight. g-C_3_N_4_ in particular has been studied for virus disinfection in drinking water [261,265,266]. Likewise, layered BP has been explored in composite photocatalysts for water treatment: when coupled with TiO₂ or other semiconductors, BP can extend light absorption into the visible range and facilitate ROS generation to destroy waterborne pathogens [33,75]. Although disinfection in water often targets bacteria, recent tests with viral surrogates (like bacteriophages) confirm that 2D photocatalysts can neutralize viruses efficiently, especially when engineered as heterostructures to maximize ROS yield [212,242]. Taken together, these examples illustrate that a variety of 2D materials have been successfully applied or prototyped in real-world antiviral contexts ranging from air and water sterilization modules to self-cleaning surfaces and personal protective equipment.

### 5.2. Environmental stability and degradation pathways

A critical consideration for deploying 2D materials in operational environments is their chemical and structural stability under working conditions. Many 2D photocatalysts undergo oxidation or phase transformation upon prolonged light exposure or air/moisture contact, which can alter their antiviral performance over time. For example, MXenes such as Ti_3_C_2_T_*x*_ are prone to gradual oxidation into TiO_2_ in the presence of water and oxygen which is further accelerated under UV illumination [267]. While the *in situ* formation of TiO_2_ could retain some photocatalytic activity, the loss of the MXene’s unique conductivity and surface chemistry can diminish the overall performance and longevity of the material. BP is even less stable. BP nanosheets readily react with ambient oxygen, humidity and light to form oxides and phosphoric acid, leading to rapid degradation of its structure [268]. In practical terms, an unprotected BP-based photocatalyst may have a limited service life, of the order of hours to days in air, unless encapsulated or modified to slow down its oxidation. Strategies like adding antioxidants, surface coatings or using red phosphorus composites are being researched to improve BP’s environmental stability [268,269].

Even nominally more robust 2D materials exhibit some degradation pathways under photocatalytic conditions. Graphitic carbon nitride (g-C_3_N_4_), for instance, can undergo photochemical breakdown of its polymeric network under prolonged UV or high-intensity visible irradiation [270]. This manifests as a loss of photocatalytic efficiency over repeated disinfection cycles, as the material may undergo deamination or carbonization, especially at defect sites. However, g-C_3_N_4_ is generally considered more stable than many metal-based catalysts, and its degradation is slower under gentle visible light, making it relatively durable for long-term use [270]. Graphene-based materials (graphene, graphene oxide, etc.) are chemically stable in typical environments. Graphene’s graphitic carbon lattice does not oxidize or decompose easily. However, functional graphene derivatives can change. For example, graphene oxide may be partially reduced by UV exposure or chemical environments, altering its surface oxygen groups and potentially its antiviral efficacy [271]. Ensuring stability might involve periodic regeneration (re-oxidation of graphene oxide) or using more stable reduced graphene as a conductive support for other photocatalysts [272]. h-BN is another 2D material known for its excellent thermal and chemical stability (often termed ‘inert graphene’). h-BN does not oxidize appreciably under ambient conditions or moderate UV, which is advantageous for longevity [273]. Its wide band gap, however, means pristine BN is not photocatalytically active under visible light [34].

For TMDs like MoS_2_ and WS_2_, stability is intermediate. The basal planes of MoS_2_ are relatively inert and can endure long illumination, but the reactive edge sites are susceptible to photo-oxidation [274]. During photocatalytic operation in oxygenated water, MoS_2_ edges can gradually oxidize to MoO*ₓ* or release sulfate species, especially under UV or blue light photons that exceed the band gap. Multilayer MoS₂ tends to corrode faster at edges than monolayers, partly due to internal layers and defects serving as additional reaction sites [274]. Other TMDs follow similar patterns: e.g. WS_2_ can form WO_3_ at edges under illumination [275]. In all cases, balancing the operational conditions (light spectrum, presence of radical scavengers, etc.) can mitigate rapid degradation.

Nevertheless, understanding and improving environmental stability remains key to practical deployment, ensuring that antiviral activity is maintained over the intended lifetime of the filter or coating.

### 5.3. Health and safety considerations

When applying 2D nanomaterials for antiviral purposes, careful attention must be given to their health and safety profiles, both for human users and the broader environment. Human exposure to these materials, specially via inhalation of aerosolized nanoparticles or wearing of 2D-coated personal protective equipment (PPE) is a primary concern. Inhaled nano-TiO_2_ is known to cause pulmonary inflammation [276]. Thus, while TiO_2_ photocatalysts effectively deactivate viruses, chronic inhalation of TiO_2_ dust (for instance, from a worn-out air filter) could pose long-term lung disease risks. Graphene-family materials present a complex toxicological profile. Pristine graphene is largely inert, but GO and other oxidized forms can interact strongly with biological tissues. Encouragingly, a recent controlled trial in humans showed that acute inhalation of GO nanosheets at low concentrations caused no observable acute pulmonary or cardiovascular effects in healthy volunteers [277]. There were no changes in lung function or major inflammatory markers after short-term exposure, suggesting that, at least in the short term, certain graphene-based antiviral coatings might be used without immediate toxicity. However, chronic or repeated exposure may tell a different story—animal studies report that inhaled GO can trigger a pro-inflammatory response in the lungs, with some impairment of the alveolar barrier and cellular stress pathways upon repeated dosing [278]. This indicates a risk of cumulative lung damage or fibrosis if graphene materials were regularly inhaled over long durations. Consequently, engineering controls (like ensuring graphene or TiO_2_ coatings do not shed respirable particles) and thorough inhalation toxicology studies are needed before wide deployment in air-filtration or PPE products.

Beyond inhalation, systemic and dermal safety of these 2D materials is also crucial. Many antiviral applications involve coatings on surfaces or fibres that could come into contact with skin or even be ingested in trace amounts (e.g. if water filters leach particles). In this regard, some 2D materials show promising biocompatibility in laboratory studies. MXenes are a notable example. Initial *in vitro* and *in vivo* tests have found MXenes to be relatively non-toxic. Ti_3_C_2_T_*x*_ MXene, for instance, caused no significant developmental or neurotoxic effects in a zebrafish embryo model up to concentrations of 100 μg ml^−1^, placing it in the ‘practically non-toxic’ category by standard scales [262]. Similarly, mice injected with MXene-based composites showed no acute toxicity or organ damage in short-term studies. These findings, along with cell culture experiments indicating minimal cytotoxicity at antiviral-effective doses, suggest that MXenes can be used in biomedical or environmental applications with a reasonable safety margin. However, it should be noted that long-term accumulation of MXene particles (or their degradation products, like TiO_2_) in organs has not been fully studied yet. Their antiviral performance must be weighed against unknown chronic effects (e.g. persistence in lungs or triggering of immune responses over time).

Graphitic carbon nitride (g-C_3_N_4_), composed of carbon and nitrogen, is generally viewed as a benign material—one report highlights that g-C_3_N_4_ showed no inherent cytotoxic behaviour in mammalian cell lines, supporting its safe use in water treatment without significant health risks [279]. Likewise, hexagonal BN is chemically inert and has been used even in cosmetic formulations. Nanoscale BN seems to have low acute toxicity, though high-aspect-ratio BN forms (nanotubes or nanofibres) can behave like asbestos and induce lung lesions if inhaled in large amounts [280]. Therefore, while BN nanosheets in a coating are probably safe due to their stability and low reactivity, any fibrous or airborne BN by-product should be avoided.

Another angle is the eco-toxicological impact of deploying 2D nanomaterials at scale. If these antiviral coatings and filters succeed, they will inevitably introduce nanoscale solids into waste streams (e.g. through water filter backwash, or as wear debris from painted surfaces). The fate of 2D materials in the environment and their effect on ecosystems is still an emerging research area. Studies of TiO_2_ nanoparticles in the environment show low acute toxicity to aquatic organisms, but chronic exposure can lead to sub-lethal effects on growth, reproduction, or behaviour of those organisms. Analogously, graphene oxide in water can interact with algae, fish or beneficial microbes; while not acutely poisonous, it may cause oxidative stress or accumulate in organisms over time. Some 2D materials might biodegrade in environmental conditions. For example, MoS_2_ nanosheets slowly oxidize and dissolve into soluble molybdate species. In fact, a recent study found that after a single exposure in mouse lungs, MoS_2_ induced an initial inflammation that was largely resolved within a month as the MoS₂ transformed into biodegradable forms and was cleared by macrophages [281]. This inherent degradability could mean less long-term persistence in the environment compared with more inert materials like TiO_2_ or graphene. On the other hand, degradation products (e.g. heavy metal ions from dichalcogenides, or phosphate from BP) could have their own toxicity. Nanomaterial leaching is a common concern in water applications. If a filter releases even a few ppb of, say, Cd^2+^ or Pb^2+^ from a doped photocatalyst, it could contaminate the treated water. Thus, material selection and stabilization are key to developing more biocompatible 2D materials for practical antiviral applications.

## 6. Perspective and outlook

In conclusion, the emerging field of 2D photocatalytic nanomaterials for the effective inactivation of viruses and bacteria holds immense promise in addressing the global challenges of infectious diseases and waterborne pathogens. This review emphasized the remarkable properties and versatile applications of these nanomaterials, demonstrating their potential to revolutionize disinfection and antiviral strategies. However, it is essential to acknowledge that our current understanding and use of 2D photocatalytic nanomaterials in this arena are still in their nascent stages.

Looking ahead, it is critical to pursue several avenues to realize the full promise of these materials. There is a pressing need to enhance antimicrobial efficacy by refining the design and synthesis of 2D nanomaterials, where adjustments in band-gap and surface characteristics could lead to superior performance. The exploration of broad-spectrum activity against a diverse array of pathogens, including those resistant to drugs, is paramount. This could be achieved through the development of versatile nanocomposites capable of simultaneously targeting various microbes.

Moreover, assessing the safety and environmental ramifications of these nanomaterials is imperative to ensure their benign integration into practical settings. Concurrently, efforts must be directed towards scaling up production to enable their widespread application, necessitating the exploration of cost-effective and scalable synthesis techniques. The translation of laboratory-scale successes to real-world applications poses a significant hurdle, necessitating collaborative efforts across the scientific community, industry and regulatory frameworks to foster innovation and safe adoption. Furthermore, the investigation of these materials’ biocompatibility is also essential for their potential *in vivo* applications.

To systematically address these challenges, future research should follow a structured roadmap. In the immediate term, efforts should prioritize establishing standardized antiviral testing platforms featuring tiered viral panels and developing *in situ* ROS probes calibrated specifically for sub-100 nm viral targets. In the medium term, emphasis should shift toward optimizing Z-scheme heterostructures compatible with indoor LED lighting, alongside comprehensive chronic inhalation and aquatic ecotoxicity assessments for flagship materials such as MXene and BP. In the longer term, integrating self-powered multilayered 2D photocatalytic stacks into HVAC systems and point-of-use water purification modules will become essential. Pilot trials conducted in healthcare settings and disaster-relief scenarios will then critically evaluate real-world efficacy and practicality.

As a consequence, interdisciplinary collaboration is vital for unlocking the expansive potential of 2D photocatalytic nanomaterials, bringing together experts from material science, chemistry, biology and engineering. Such collaborative efforts are essential for driving forward the innovative solutions and breakthroughs in this domain. Additionally, the COVID-19 pandemic has highlighted the critical need for advanced antiviral strategies, positioning research in 2D photocatalytic nanomaterials as a cornerstone for future global health and pandemic preparedness.

In summary, the use of 2D photocatalytic nanomaterials for virus and bacteria inactivation represents a cutting-edge and transformative approach. While challenges remain, the prospects are bright, and continued research and innovation will probably pave the way for the development of effective, sustainable and scalable solutions in the fight against infectious diseases and virulent pathogens. This evolving field offers hope for a healthier and safer future, emphasizing the importance of ongoing exploration and application.

## Data Availability

All the data are from published articles and cited appropriately.
